# Gepoclu: a software tool for identifying and analyzing gene positional clusters in large-scale gene expression analysis

**DOI:** 10.1186/1471-2105-12-34

**Published:** 2011-01-26

**Authors:** Tania Dottorini, Nicola Senin, Giorgio Mazzoleni, Kalle Magnusson, Andrea Crisanti

**Affiliations:** 1 University of Perugia, Department of Experimental Medicine, via del Giochetto, Perugia 06100, Italy; 2 University of Perugia, Department of Industrial Engineering, via G.Duranti, Perugia 06125, Italy; 3 Imperial College London, Biological Sciences, Imperial College Road, London SW7 2AZ, UK

## Abstract

**Background:**

The notion that genes are non-randomly organized within the chromosomes of eukaryotic organisms has recently received strong experimental support. Clusters of co-expressed and co-localized genes have been recognized as playing key roles in a number of functional pathways and adaptive responses including organism development, differentiation, disease states and aging. The identification of genes arranged in close proximity with each other within a particular temporal and spatial transcriptional program is anticipated to unravel possible functional links and reciprocal interactions.

**Results:**

We developed a novel software tool *Gepoclu *(Gene Positional Clustering) that automatically selects genes based on expression values from multiple sources, including microarray, EST and qRT-PCR, and performs positional clustering. *Gepoclu *provides expression-based gene selection from multiple experimental sources, position-based gene clustering and cluster visualization functionalities, all as parts of the same fully integrated, and interactive, package. This means rapid iterations while exploring for emergent behavior, and full programmability of the filtering and clustering steps.

**Conclusions:**

*Gepoclu *is a useful data-mining tool for exploring relationships among transcriptional data deriving form different sources. It provides an easy interactive environment for analyzing positional clustering behavior of co-expressed genes, and at the same time it is fully programmable, so that it can be customized and extended to support specific analysis needs.

## Background

Genome-scale gene transcription analyses obtained from microarrays and EST studies generate large datasets that must be adequately processed to extract valuable functional information. This can be best achieved by detecting a particular pattern or behavior in the properties of the genes in term of sequence, transcription profile and chromosomal location to unravel functional relationships which otherwise would pass unnoticed. *Gene clustering *is a statistical process aimed at collecting genes into logical groups (clusters), based on the observed similarity of one or more elements or properties that can be ascribed to such genes. In particular, *positional clustering *aims at identifying groups of genes characterized by *positional proximity *within a chromosome. The rationale for investigating positional clustering in expression dataset is twofold: first it facilitates the organization of large amount of information into higher-level structures which could shed new light on less visible properties of the dataset; second it allows the identification of co-expressed and co-localized genes that could be functionally and/or evolutionary linked. In several eukaryotic organisms non-homologue but functionally linked and co-transcribed genes have been reported to cluster together in physical proximity unraveling a gene organization with operon-like features, i.e. co-regulation of neighboring genes [1]. In particular, several lines of evidence have demonstrated that proximity of co-expressed genes does not occur at random, but rather underlies a selection process involving genes working together in signaling and metabolic pathways [1-6]. Non-random organization and gene clustering have clearly emerged from genome-wide analysis as biological relevant phenomena [4,7-22]. The importance of gene positional clustering analysis is also widely recognized, but only few informatics tools are available to support the analysis of large-scale gene datasets [10,23-25]. Furthermore, discrepancies between approaches and interpretations of results have been observed [5,16,26] as well as lack of cross-references to prior results [5]. Widespread methodologies and bioinformatics tools of general applicability are increasingly needed to support this type of analysis.

In this work, a method of general applicability implemented by a suite of software applications (*Gepoclu *or Gene Positional Clustering) is presented, that performs positional clustering analyses of genes automatically preselected based on expression data obtained from microarray, EST, qRT-PCR and PCR experiments. Large amounts of information can be easily processed and clustering results can be visualized and or further processed with different types of quantitative analysis. *Gepoclu *is also capable to take into account *multiple expression values *associated to the same gene to support comparative analysis scenarios where the same gene may be expressed multiple times as for example in different developmental stages of an organism, during aging, in distinct organs/cell types, unhealthy and disease conditions, in infected and non-infected, and so forth.

## Implementation

### *Gepoclu *software architecture

**Figure 1:**
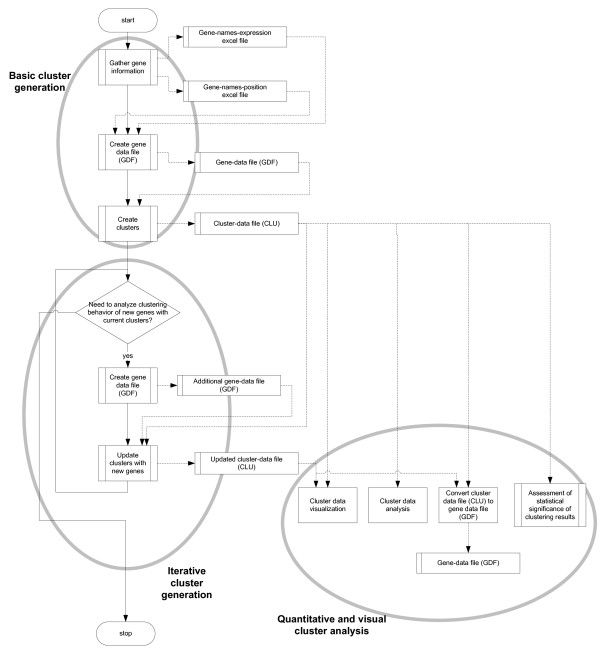
**Proposed procedure for positional clustering analysis with *Gepoclu***. The flowchart illustrates the main steps of the procedure along with the produced data files. Most of the flowchart boxes represent top-level processes comprised of several additional sub-processes. A detailed run down of the entire procedure is illustrated as additional flowcharts in the Additional data file 1.

*Gepoclu *is essentially a collection of Matlab functions (a library of functions) that can be embedded in user applications that address gene positional clustering problems. Furthermore, *Gepoclu *provides a suite of interactive software applications, based on the same functions, which can be launched from within the Matlab programming environment, and allow non-programmer users to make use of the same functionalities through a console-based textual interaction mechanism. Twelve interactive applications are currently available for immediate use, all designed to read/write data from/to specially formatted, user-readable text files; mostly suitable to be used in any order, depending on user needs. By directly accessing the *Gepoclu *library of functions, it is possible to create additional applications that automate specific analysis processes and/or fulfill more specific analysis needs. A standard analysis methodology that is based on already available applications has been documented in the Results section, and in Figure 1. Further details of the entire analysis process and functionality of the suite of applications are provided in the Additional material (Additional file 1). The entire *Gepoclu *library of interactive and non-interactive functions is provided in the Additional file 2. The contents include a fully automated script and all the data needed to replicate the most relevant results of the example application 3.

### Portability and interoperability

*Gepoclu *operates on any operating system and hardware platform that supports Matlab, including MS Windows, Apple OS, the main flavors of Linux and Unix. No additional Matlab libraries or toolboxes are required for *Gepoclu *to work. All the interactive applications provided by *Gepoclu *accept input data from text files and provide output as text files. The file formats used by *Gepoclu *(gene data file - GDF and cluster data file - CLU) are user-readable/writable and can be easily shared with third-party applications through the implementation of simple read/write filters. For implementing a specific functionality (i.e. merging gene positional and expression data), MS Excel files are currently required as input; however, support for plain text input for this phase is planned as part of future development. Vector-graphics images generated through *Gepoclu *(multiple-clusters view and single-cluster view) are provided as Matlab figures, and therefore rely on Matlab to be converted into any of the supported raster or vector-graphics formats (Adobe illustrator, Bitmap, EPS, Enhanced metafile, JPEG, paintbrush 24.bit, portable bitmap, portable document format, portable gray map, portable network graphics, portable pixmax, portable in Kmap, TIFF, TIFF no compression file, etc.).

### Testing

We have used and tested *Gepoclu *on typical Windows and Mac OsX PCs (2-GHz processor, 4 GB of memory). A clustering analysis performed on an eukaryotic genome such as *Anopheles*, and containing thousands of genes, and with a high cluster density (about 400 clusters spread on all chromosomes) takes a little less than one minute, simpler data sets can be analyzed in a few seconds (this specifically refers to clustering time, data loading and preprocessing may take longer depending on the size of the initial data set). The visual representation of about 400 clusters on chromosomes with activated labeling mode for cluster ID and base pair positioning within chromosomes, takes less than 10 seconds. *Gepoclu *has been applied to variety of data sets from different sources concerning both genomes and expression data.

### Interactive *Gepoclu *applications

In this section, the twelve main interactive applications provided by *Gepoclu *are listed. As stated earlier, these applications provide a simple console-based interactive user interface and encapsulate the functionality provided by the complete *Gepoclu *library of functions. Interactive applications can be recognized as their names start with "i". An example of using Gepoclu through non-interactive functions (i.e. through a fully automated script) is also provided in Additional file 2.

#### iGdfCreator

This application merges the two Excel files containing gene positional and expression information. During merging, duplicate genes are identified (based on name) and removed, while warning messages are generated. In duplicate removal, only the first encountered instance of a gene is kept. The resulting information is formatted as a text-based, gene data file (GDF); the names of the duplicate genes are written to a separate text file.

The duplicate-removal functionality has been designed into *Gepoclu *mainly for handling replicates, which may have been unintentionally left by the operators in the input files. *Gepoclu *does not directly handle the problem of deciding how to manage multiple probes of a gene. This was a precise design choice as there may be several different approaches to handling duplicates depending on peculiar aspects of each application: sometimes an average should be computed, other times subsets may be recognized as outliers and discarded accordingly, other times measurements should be discarded altogether and entirely repeated due to excessive dispersion of data. To avoid unnecessary risks involved in automating decisions related to duplicate handling, *Gepoclu *is limited to generating appropriate warnings and to providing a minimal solution (i.e. keep the first instance of any replicate gene) to ensure the entire analysis process could be completed anyways in case the operator may decide against taking corrective actions.

The two Excel files must be formatted so that they conform to the following requirements:

Expression file (.xls)

genename expr1 expr2 expr3 expr4 .....

genename expr1 expr2 expr3 expr4 .....

.......

Position file (.xls)

genename chrom start end strand

genename chrom start end strand

...

If positional clustering is to be done regardless of gene expression data information, an expression file with expression values set to zero must be created. Names in the expression and position files must correspond to the same genes. The expression file supports multiple gene expressions, missing expression values are replaced by *NaN *(not a number) strings at merging. Expression values may be provided as ratios, normalized, logarithmic values, depending on the experiment (microarray, qRT-PCR, EST, PCR) or in any other user-defined format, as long as all the values are defined in the same reference so that they can be compared. The position file must contain all the required information for each gene: chromosome name, start and end in base pairs, and strand (encoded as ±1). The required format for the position file is consistent with the output format provided by the Biomart database. Merged gene information is written to a text file in the GDF (Gene data file) format. The format is defined as follows:

Gene data file (.gdf)

dset genename chrom start end strand expr1 expr2 expr3 expr4 .....

dset genename chrom start end strand expr1 expr2 expr3 expr4 .....

.....

Where the only additional information is a reference name for the dataset (*dset*, sometimes also called *origin*), provided by the user during the interactive merging session, and which is useful when multiple GDF files must be used in iterative clustering investigation processes (see also later). Genes listed in the GDF files are listed in properly sorted order, according to position on each chromosome.

#### iGdfNaNFixer

This application amends a GDF file by replacing *NaN *expression values with a value provided by the user. The fixed file is saved as a new text file, in the GDF format.

#### iGdfFilter

This application is used to discard/keep genes based on a threshold defined on expression values. The input is a GDF file and the output is another GDF file containing only the genes that passed the threshold criterion. To allow for testing multiple expression values against the same given threshold, expression indices can be provided to determine what expressions will undergo testing; this way, either one, many or all expression values can be considered when filtering genes.

#### iCluCreator

This is the main function for creating positional clusters from a set of genes, which must be provided as a GDF file.

Each chromosome is processed separately. Clusters are formed according to the following rule: the gene currently under consideration belongs to the same cluster of the previous one if the distance between the two gene starting points is smaller or equal than a given distance threshold, defined as a multiple of one base-pair (bp), regardless of the strand. If the distance is larger than the threshold, the current gene becomes the first one of a new cluster. As clusters are formed, they are tagged with a progressive, numeric identifier. Clusters formed by only one gene are eliminated during chromosome traversal. The distance threshold is provided (in bp) by the user during the interactive analysis session.

No expression information is used during clustering. It is assumed that any expression-based filtering has already been done in the previous steps.

Cluster information is stored in a text file written in the CLU (cluster file) format, which is structured as follows:

Cluster file (.clu)

nClusters N

1

beginCluster

dset genename chrom strand start stop exp1 exp2 ...

dset genename chrom strand start stop exp1 exp2 ...

dset genename chrom strand start stop exp1 exp2 ...

......

endCluster

2

beginCluster

dset genename chrom strand start stop exp1 exp2 ...

dset genename chrom strand start stop exp1 exp2 ...

........

The total number of clusters (N) is written at the beginning of the file, the clusters are listed in order, preceded by their numeric identifier and delimited by a set of begin-end statements. Within each cluster, the list of contained genes is reported, with complete positional and expression information.

#### iCluAdder

This application offers the possibility to update current clusters with new gene datasets. It is used to identify the behavior of a set of genes when added to existing clusters. The new genes may be incorporated into existing clusters, or they may form new independent clusters. The rules for creating clusters are the same used in iCluCreator and it is recommended that the same distance threshold parameter is used.

The application operates with input data coming from an existing CLU file and a new GDF file containing the additional genes to be processed, the updated clusters are stored into a new CLU file. To be able to distinguish between the "original" genes and the newly added genes in the new CLU file it is convenient to tag the additional genes with a different dataset name in the new GDF file: a typical cluster containing added genes will look as follows in the updated CLU file:

beginCluster

dset genename chrom strand start stop exp1 exp2 ...

dset genename chrom strand start stop exp1 exp2 ...

dset genename chrom strand start stop exp1 exp2 ...

dset2 genename chrom strand start stop exp1 exp2 ...

dset2 genename chrom strand start stop exp1 exp2 ...

......

endCluster

#### iCluToGdf

This application allows for extracting a single cluster from a CLU file and saving its contents (list of genes) as a GDF file.

#### iClusterDisp

This is a utility application that displays on screen the contents of a user-selected cluster located within a CLU file.

#### iGeneFind

This is an utility application that searches for the gene in the clusters, and returns the numerical identifier of the cluster containing the gene (if any).

#### iClusterView

**Figure 2:**
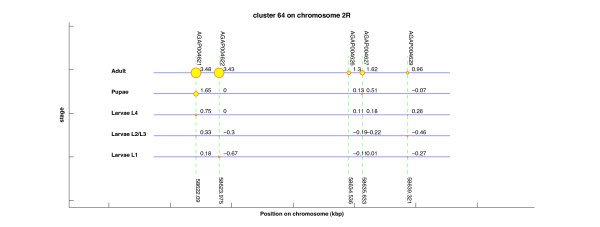
**Example of single-cluster visualization obtained from *Gepoclu***. Gene data refers to one of the clusters computed in the example application 3, described in the text. In the vector graphics representation label positions were manually adjusted to enhance readability.

This application allows for selecting a single cluster from a CLU file and generates a vector-graphics representation that is visualized in a Matlab figure. The representation of the cluster is known as single-cluster view, as it was illustrated in the Results section (Figure 2).

#### iAllClustersView

**Figure 3:**
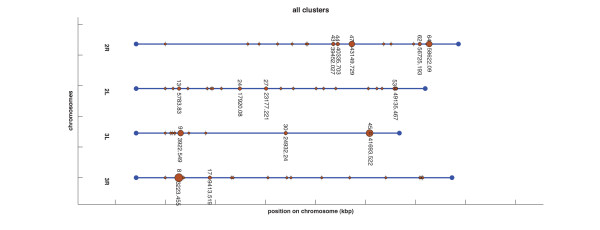
**Example of multiple-cluster visualization obtained from *Gepoclu***. Clusters generated as described in the example application 3, reported in the text. In the vector graphics representation label positions were manually adjusted to enhance readability.

This application allows for visualizing all the clusters contained in a CLU file in a proper vector-graphics format. The format was referred to as multiple-clusters view and was illustrated in the Results section (Figure 3).

#### iRndCluCreator

This application runs the positional clustering analysis onto sets of randomly selected genes. It is useful for generating data to be used in statistical tests aimed at evaluating the statistical significance of the clustering results obtained for the set of genes being scrutinized.

To produce random clustering data to be used for comparison with actual clustering results for a given set of n selected genes, a GDF file must be prepared containing a larger set of genes (which may or may not include the selected ones). The application reads n genes randomly from the GDF file and runs the clustering analysis. The entire process (extracting *n *randomly selected genes and running the clustering analysis) is repeated automatically a number of times defined by the user, in order to collect sufficient information for the random sample. Once the process is finished, final clustering data are aggregated and provided as a text file, for being processed, along with the "real" clustering results in third-party statistics software applications, or within Matlab itself.

The text file (see Table 1) is formatted as in the following example:

**Table 1:** **Example output file as produced by iRndCluCreator**

**run**	**n.clusts**	**n.tot.clust.genes**	**n.genes per cluster**
1	3	6	2 2 2
2	3	7	2 3 2
3	3	6	2 2 2
4	1	2	2
5	3	7	2 2 3
6	2	4	2 2
7	5	13	2 3 2 4 2

To read the file, consider for example the fourth row, which refers to the third run (run:3). Three clusters were generated (n.clusts: 3), a total of 6 genes were associated to clusters (n.tot.clust.genes: 6) and each cluster contained exactly 2 genes (n.genes per cluster: 2 2 2).

#### iAllClustersAnalyze

This is a simple utility function that provides quantitative information concerning the contents of a CLU file. More specifically, it returns the probability distribution of cluster sizes, written as a text file.

### Methods applied in the example applications for *Anopheles gambiae*

Microarray expression data for the example application n.1 (clustering analysis for blood-fed and non blood-fed adult, female mosquitoes) was taken from previous literature work [27]. Non-quantitative PCR data for the example application n.2 (clustering analysis of male accessory gland genes) was also taken from previous literature work [28]. Microarray expression data for the example application n.3 (clustering analysis of male and female mosquitoes at different developmental stages) was taken from unpublished material (K Magnusson, unpublished, but see Additional file 3 and Additional file 4). In all three example studies, *Anopheles gambiae *gene positional data was extracted from Biomart, using gene identifiers to retrieve chromosome names, start and end positions (in bp) and strand. For some genes it was not possible to extract coordinates from Biomart: those genes were excluded from the computations. The positional information for the

*Drosophila melanogaster *Acp genes (used in the example application n.2) was retrieved from Biomart as well.

Positional distance thresholds used to create clusters were computed from mean gene density information retrieved from the literature [29-31] and from online resources (http://www.ensembl.org and http://www.vectorbase.org).

For assessing the significance of any clustering result obtained with the selected genes, with respect to the clustering of a random set of genes, *Gepoclu *was used to randomly extract sets of genes and generate clusters, and a third-party statistical software application (SPSS) was used to run the appropriate statistical tests (generally, the one sample student's t-test; see Additional files 5, 6 and 7).

## Results and Discussion

### Software functionality

*Gepoclu *is a collection of software tools that functions in two ways: either as an interactive console-based applications run from within the *Matlab programming environment *[32], or as a set of Matlab programmable functions that can be embedded in a wide array of user-defined software applications. *Gepoclu *has been designed to be operated according to a predefined analysis protocol, that can be summarized into three main steps: basic cluster generation, iterative cluster generation and quantitative/visual cluster analysis (Figure 1, and Additional file 1); the protocol includes a preliminary activity that must be carried out manually by the operator, limited to initial data acquisition; the remaining activities defined in the protocol are *computer-assisted*, meaning that they are designed to be carried out with the support of dedicated *Gepoclu *data analysis functions. The main steps of the protocol, and related *Gepoclu *functionality are described in the following.

#### Assembly gene information (manual operation)

*Gepoclu *accepts gene position data information from online resources, such as *Biomart *http://www.biomart.org that include *chromosome *or *contig*, gene *start*/*end *points (expressed in base-pairs) and *strand *information. The software also accepts gene expression information (i.e. transcription regulation) from experiments involving microarray, EST, qRT-PCR or PCR analysis, or from online data collected from previous research activities. Gene positional information is essential for clustering analysis, while gene expression data can be omitted; in such a case cluster analysis will be performed taking into account only positional data. *Gepoclu *is capable of processing different expression values for large sets of genes, originated from different developmental stages, cell differentiation, or obtained under different experimental conditions. As a general rule, expression data should be formatted in numerical values in the same reference scale. In case of multiple expression data, it should be also ensured that the same number of expression values is available for each gene. Following the protocol, positional and expression data must be manually collected into two properly formatted *Excel *files (see Additional file 1 for the format requirements). The format of the Excel file containing positional information has been designed to allow for the insertion of Biomart-formatted data with the minimum conversion effort.

#### Integrating positional and expression information (computer-assisted)

*Gepoclu *integrates expression and position information into a single, coherent data structure, properly formatted for downstream data processing analyses. In this step, gene duplicates (if any) are identified and removed, together with inconsistencies in the input data sets (e.g. missing expression information, missing positional information). Furthermore - if required by the specific analysis process - genes may undergo a filtering process based on expression levels, which may mean either to exclude genes with missing expression information (if expression is to be considered), or to exclude genes with expression levels below a user-defined threshold. Since the software supports also clustering based on positional information alone, expression information can also be omitted altogether. The integration of positional and expression data is carried out by *Gepoclu *in a fully automated manner, though some parameters may be set out by the user to investigate the effect of different variables (e.g. filtering genes based on expression threshold value) thereby facilitating the processing large amounts of gene data with ease. The output of this step of the procedure is a single file containing merged information (positional information and one or multiple expression values) for all the genes of the analysis; genes are ordered according to their position in the chromosome they belong to. Notice that at this point the list of genes may differ from the initial one, as some genes may have been discarded because of missing information, or because of not fulfilling the threshold requirements. The merged information is saved as a text-based file known as the *gene data file *(GDF): the file is formatted so that it is compatible with *Gepoclu*, however, the format is readable by the operators and may be also imported into third-party data processing applications.

#### Generation of positional clusters (computer-assisted)

This step is carried out with full support of *Gepoclu*, functionalities starting from the *gene data file *(GDF) and analyzing one chromosome at a time. Within the same chromosome, genes are processed sequentially in the order they are defined in the file. This is because the genes were initially sorted according to their position in the chromosome. At this stage, all genes in the GDF are analyzed irrespectively of their expression values since they had already been filtered through the expression threshold level (see previous step). Clusters are generated according to the following rule: if two genes have start sites whose distance - measured in base-pairs (bp) - is smaller or equal than a given *distance threshold*, regardless of the strand, then they belong to the same cluster. Distance threshold is the only parameter that must be decided by the operator depending on the application, and is generally related to mean gene density of the analyzed genomes. Isolated genes (i.e. distant from the previous and next genes by more than the threshold) are discarded, since single-gene clusters are not allowed. The output, at this stage, is a list of clusters for each chromosome: each cluster is labeled with a numeric identifier and contains a list of genes. Cluster information is stored in a text file written in proprietary format, known as the *cluster file *(CLU).

A few notes on the rationale behind this step. First of all, in Gepoclu, clustering is done by defining a distance metric based on gene position alone. Therefore, in a strict sense, expression values are not used at clustering. Gene expression values are used for selecting what genes to include in the clustering process. The main advantage offered by *Gepoclu *with respect to other clustering packages is to provide both expression-based filtering, and position-based clustering functionalities as parts of the same fully integrated, and interactive, environment. This means rapid iterations while exploring for emergent behavior, and full programmability of the filtering and clustering steps.

A second consideration relates to, strand information not being used when forming clusters. This comes from previous literature findings confirming that genes in adjacent or nearby positions along the chromosome influence the transcription of each other, regardless of their orientation, i.e. of the strand (forward or reverse) they are located into [18,33-35]. For this reason, *Gepoclu *uses only the positional information (in bp or kbp) as obtained by major databases, such as Biomart.

In any case, it is still possible to take into account the strand when running analyses with *Gepoclu*, by preparing lists of genes that specifically belong to one strand only. In this case, a proper query must be made in the genomic database to filter out genes not belonging to the strand under investigation.

A third note is related to the choice of adopting a single distance threshold value. In real-life applications average gene length and density are often genome-specific, and can also vary within the same chromosome, therefore the use of a single threshold value, constant throughout the entire length of the chromosome region being analyzed, may appear as a limiting choice. However, a single distance metric, measured either in bp or kbp, was believed as being more understandable by end-users and easier to relate to when setting the goals of clustering analysis in specific application domains. In particular, setting the distance threshold depending on mean gene density was found as a sound choice in most cases. Conversely, if application-specific analysis goals make adopting a single threshold value insufficient, multiple additional clustering analyses can be run with different thresholds: this in particular allows for investigating how regions at different densities react in terms of clustering propensity.

#### Visual representation of clustering results (computer-assisted)

*Gepoclu *provides functions for the visual display of the clustering results in original graphical forms, to facilitate the identification of the most relevant aspects of cluster distribution and composition, and ultimately, with the aim of inferring novel information from available data. Clustering results, rendered in graph-based forms, can be visualized directly within the Matlab programming environment, edited and saved into any of the most common raster and vector-graphics image formats. Several examples of data display generated using *Gepoclu *are shown in this work (Figure 2 and 3): albeit most of the visual result has been generated automatically in such figures, a few colors and caption positions have been manually edited to enhance clarity. We have properly indicated manual edits in the figure legends, to clearly highlight the actual output capabilities of *Gepoclu*. The software provides two main *visualization modes*: one, referred to as "multiple-cluster view", that shows the relative position and the size of individual clusters along the chromosome; the other, referred to as "single-cluster view", that shows the contents of single clusters. The multiple-cluster view is designed to display in visual form information concerning the number of clusters on each chromosome, their reciprocal position and how populated they are (i.e. how many genes are within each cluster). As an example of multiple-cluster view (discussed in more detail in the following paragraphs) we have analyzed the positional clustering of a set of genes that were selected on the basis of their temporal and spatial transcription profile in adult *Anopheles gambiae *male mosquitoes, through microarray analysis (Figure 3). Each chromosome is represented as a horizontal line; there are as many horizontal lines as many chromosomes were listed in the original gene data file (four chromosomes, in Figure 3). Each chromosome is not drawn for its entire length: only the region going from the first (leftmost) cluster to the last (rightmost) cluster is plotted. All chromosomes are left-aligned in the figure with reference to their relative lengths. Chromosome lines are labeled with the actual chromosome names, as reported in the GDF. Each cluster is represented as a colored circle. Diameter is proportional to how populated is the cluster, thus larger circles indicate larger clusters. Clusters are labeled with a number, which is the identifier assigned to the cluster at creation (see previous step). The identifier allows also for the recognition of the cluster in the CLU file. Each cluster is also flagged with a label that indicates the position (in kbp) of the first gene of the cluster. For large data sets, this kind of visualization can generate quite a complex display. However, the figure is entirely generated by *Gepoclu *in vector-graphics format, therefore the position of the drawing primitives can be manually adjusted at any time from within the figure to reach to a more effective visualization of the result. Most of the figures illustrated in this work have undergone this manual adjusting process, as indicated in their captions. An example of the second main visualization mode, referred to as "single-cluster view" is also illustrated (Figure 2). The figure shows one of the clusters identified in Figure 3 (cluster 64 on chromosome *2R*). The aim of the single-cluster view is to visualize the contents of an individual cluster. *Gepoclu *is capable of handling multiple expression values for the same set of genes. The information about multiple expression data associated to a cluster is visualized by showing the same portion of the chromosome where the cluster is located as a series of lines, each referring to a specific experimental conditions examined. Each line works as a placeholder for a single set of expression data values, called *stage *in the figure, and for each set, the expression value associated to each gene affects the diameter of the corresponding circle. Diamonds can be used instead of circles to visualize expression levels, to avoid any potential confusion with the circles, which are also used in the multiple-cluster view. In Figure 2 there are five expression patterns corresponding to five developmental stages of *A. gambiae; *it is clearly evident that most of the genes contained in the cluster are mainly expressed at the *adult *stage, as they are represented with circles of larger diameters. In the special case that no expression values are provided (i.e. pure positional clustering regardless of expression), the single-cluster view will show the gene contents of the cluster with circles reduced to single points (i.e. 0 expression mapped to 0 diameter). Finally, if the data utilized to generate the clustering analysis originate from different data sets, the single-cluster view will use a different color for each data set.

#### Quantitative analysis of clusters (computer-assisted)

*Gepoclu *is designed to provide support to perform statistical analyses on clustering data. The first type of analysis is dedicated to *computing quantitative indicators of clustering behavior*. Examples of such indicators may be: total number of clusters, number of clusters per chromosome, average number of genes per cluster, probability distribution of the number of genes per cluster, probability distribution of the distances between clusters, etc. Functions are provided to extract the contents of each cluster, including number of genes, and names and attributes (expressions, position) of each gene. Retrieved information is saved in text format, and can be imported into any statistics or math software package for computing the desired descriptors. The second type of analysis is dedicated to *assessing statistical significance *of the clustering result. Following the approach illustrated by Boutanaev et al. [36], the aim is to evaluate whether the positional clustering result obtained with the selected *n *genes is to be considered *statistically different *from a positional clustering result obtained with another set of *n *genes randomly selected from the same chromosomes. To this purpose, *Gepoclu *provides functions for randomly selecting genes from a GDF, while clustering of such genes can be done with the same functions illustrated before. *Gepoclu *provides the functions for exporting relevant quantitative information pertaining clusters in text format, all the data can be transferred to any statistics package (including Matlab itself) for running additional statistics tests: an example test worth mentioning is illustrated in the work by Li et al. [34]: in this case, the significance of each single cluster can be assessed in relation to local gene density.

#### Iterative investigation of gene clustering tendencies (computer-assisted)

At any moment of the analysis, new genes can be added to the initial set, to investigate whether they merge with existing clusters or form new independent ones. Updated results can be visually displayed and compared with previous results, or again subjected to quantitative analyses as illustrated previously. *Gepoclu *provides all the needed functions to implement this type of investigation, which implies an iterative trial-and-error approach aimed at identifying emergent clustering behavior. The very same nature of the entire suite of Matlab functions, designed as standalone interactive modules operating with input/output text files, supports an analysis process which may not necessarily be strictly sequential, allowing for a more free approach to positional clustering investigation.

#### Role of Gepoclu in current clustering research

As stated earlier in the background section and discussed in previous work [5], discrepancies between studies regarding the sizes and locations of clusters of co-expressed genes in the same species do exist. Different experimental and computational approaches are being adopted, and there is a paucity of literature work that systematically compares the available alternatives. While future research may greatly benefit from the development of a more uniform framework for positional clustering, for now *Gepoclu *provides just one more possible viewpoint to approach the problem. Like any other viewpoint, our solution to positional clustering highlights specific aspects of the datasets it is applied to, and such aspects may add up to other properties, which may be highlighted by different approaches. In the end, we believe that further insight into gene expression analysis problems may be gained by applying the analysis method proposed by *Gepoclu*, as illustrated for the following example applications.

### Example applications of *Gepoclu *for gene positional clustering of co-regulated *A. gambiae *genes

To illustrate the proposed methodology and *Gepoclu *capabilities we have used this software to identify co-expressed and co-localized *A. gambiae *genes involved in key behavioral processes such as feeding and mating. We have analyzed a large amount of experimental data and gene expression profiles including: (example app. 1) the genome-wide analysis of gene expression of adult *A*. *gambiae *mosquito [27]; (example app. 2) a genome-wide analysis of male accessory gland genes, possible modulators of female behavior [28]; and (example app. 3) the microarray analysis of sex biased genes expressed during early developmental stages (K Magnusson, unpublished, data provided in Additional files 3 and 4). We ran several positional clustering analyses on such data, with the intent of investigating the existence and variation of hot-spots of co-expressed and co-localized genes depending onto feeding conditions, mating, tissue specificities, and different developmental stages. These analyses provided suitable examples to demonstrate the investigation power of the proposed approach and *Gepoclu *software.

#### Example application 1: Positional clustering behavior of genes differentially transcribed in blood-fed female adult mosquitoes, at different time points - microarray data

**Figure 4:**
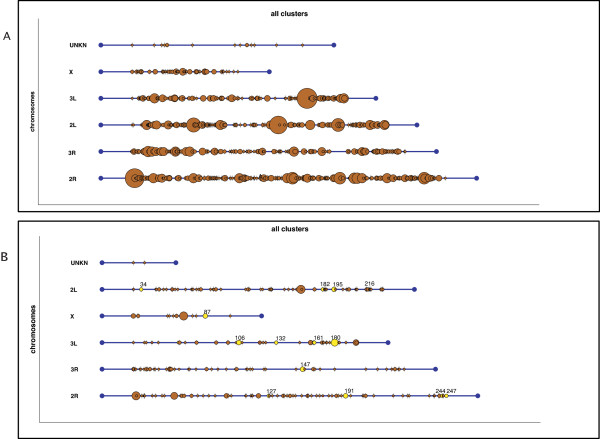
**Multiple-cluster views obtained from *Gepoclu *showing clustering behavior of the up-regulated genes of *pbf *female mosquitoes at different time points**. Computed from the microarray data available from [27] a) 3-24 hrs. up-regulated genes in *pbf *mosquitoes; b) 24-48 hrs. up-regulated genes in *pbf *mosquitoes. Special-interest clusters identified through *Gepoclu *in pbf mosquitoes were manually colored yellow.

We used *Gepoclu *to analyze the clustering of female mosquito genes transcribed in blood-fed female mosquitoes (between 3 hrs and 15 days after feeding). Microarray data was retrieved from previous literature work [27] (blood-fed mosquitoes are referred to as *pbf *mosquitoes); microarray data was formatted as a lists of up-regulated (*up*) and down-regulated (*down*) genes for *pbf *mosquitoes, obtained at different time points. For the example application, the binary expression-based classification (up/down regulation) was deemed sufficient for the analysis, and no further specific threshold were applied on gene expressions. Clustering analyses were performed separately for the *up *and *down *genes (Table 2 and Additional file 5). Positional distance threshold for cluster formation was set to 20 kbp, on the basis of the mean gene density values reported from previous investigations [30,31]. Our analysis showed that clustering behavior varied as a function of time elapsed from feeding. The clusters that were identified in the data sets were also analyzed with the statistical functions available in the software (see Table 2). The table lists the percentages of clusters populated by different numbers of genes, starting from two-gene clusters. As control for each clustering analysis performed using microarray data, a total of 20 clustering attempts where done using randomly selected genes from a list of 12457 anopheles coding sequences retrieved from Biomart; results were compared with the one sample student's t-test, in SPSS [37] (see Additional file 5). First, we used the clustering capabilities of *Gepoclu *to identify the time-frame subset (hours) and gene subset (up/down - regulated genes) where to focus the analysis for the identification of populated hot-spots of co-localized and co-expressed genes to be used as indicators of feeding. In the analysis of the data we did not take into account two-gene clusters as transcriptional hot-spots, on the assumption that many of them could be due to local duplication events [34,36], hence we concluded that conditions where two-gene clusters would coexist with larger clusters in approximately the same amounts would be less interesting for the analysis. This led us to identify the time-frame comprised between 3 hrs. and 24 hrs. after feeding of the up-regulated genes as the most interesting window of analysis, in terms of significance of the clustering result; clusters identified for the 3-24 hrs. time frame for up-regulated genes are shown in figure 4.A.

**Table 2:** Summary of cluster sizes for *pbf *mosquitoes as obtained through *Gepoclu*.

PBF UP-REGULATED	NBF-3 h UP	3-24 h UP	24-48 h UP	48-72 h UP	72-96 h UP	96 h-15 d UP
n. of clustered genes/total genes	0/1883	2609/3667	589/1422	145/542	618/1340	0/2

% of genes in clusters n = 2	NA	29.8%	61.8%	73.1%	59.2%	NA

% of genes in clusters n = >3	NA	70.2%	38.2%	26.9%	40.9%	NA

% of genes in clusters n => 4	NA	46.6%	13.2%	6.2%	20.1%	NA

**PBF DOWN-REGULATED**	**NBF-3 h**	**3-24 h**	**24-48 h**	**48-72 h**	**72-96 h**	**96 h-15 d**
	**DOWN**	**DOWN**	**DOWN**	**DOWN**	**DOWN**	**DOWN**

n. of clustered genes/total genes	856/1835	1123/2179	512/1221	133/528	1014/1824	42/179

% of genes in clusters n = 2	53.3%	41.5%	57.8%	57.1%	47.9%	47.6%

% of genes in clusters n = >3	46.7%	58.5%	42.2%	42.9%	52.1%	52.4%

% of genes in clusters n = >4	22.5%	32.6%	15.8%	33.8%	23.1%	23.8%

Clustering applied to females, up-regulated and down-regulated genes. Gene expression data from Microarray.

NBF: non-blood fed female mosquitoes

Then, we analyzed those *pbf *clusters (3-24 hrs.) that contained at least four genes, but we focused only on clusters, which were still preserved at the subsequent time frame (24-48 hrs.). As a rule we set that at least three of the genes initially present in the cluster would be maintained, and also that the cluster would never become smaller than three genes in total. This analysis yielded a total of 13 clusters (shown in yellow in Figure 4.B, for the 24-48 hrs. time frame), which deserve to be further investigated as they appear to be the only clusters continuously up-regulated within the first 48 hrs.

#### Example application 2: Analysis of clustering behavior of male accessory gland genes - non quantitative PCR data

**Figure 5:**
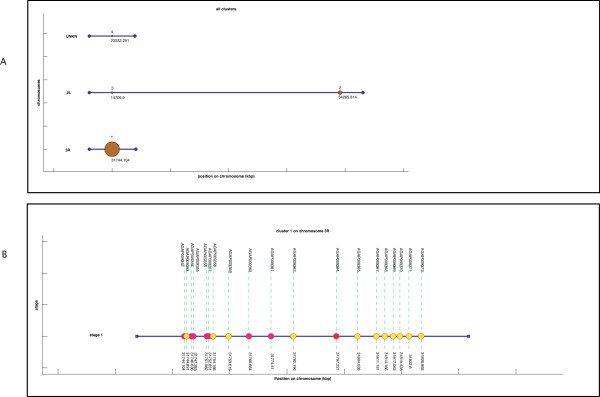
**Results of the iterative clustering obtained from 5 data sets of the male accessory gland (mag) proteins**. PCR data obtained from the five data sets indicate genes found in different tissues [28]. a) multiple-cluster view of the final result of the iterative process; b) single-cluster view of the largest cluster found on chromosome 3R. Gene expression colors indicate the coexistence of data coming from different data sets within the same cluster (yellow: *mag only*; purple: *mag and testis*; red: *mag, rest of the body, females*. Some label positions were manually adjusted to enhance readability.

The dataset is comprised of a total of 46 genes and putative alleles from an alternative haplotype (see Additional file 6) related to the male accessory gland (mag) proteins, relevant in mating, and identified through a genome-wide analysis approach in previous work by some of the authors [28]. In this work a number of genes were classified in five groups on the basis of their tissue transcription specificity, as deduced from non-quantitative PCR data: (i) *mag only*; (ii) *mag and testis*; (iii) *mag, testis and females*; (iv) *mag, rest of the body and females*; (v) *mag and ubiquitous*. Each gene data set was separately processed for clustering. The results obtained through *Gepoclu *(with 20 kbp positional threshold) showed a clustering behavior significantly different from what achievable from a random set of genes (analyzed with the same statistical approach illustrated in the previous example); in particular, the observed cluster distribution for these genes indicated a highly significant bias towards localization on particular regions of chromosome 3R (see Additional file 6). We also investigated whether the homologue mag genes in *D. melanogaster *had a biased distribution. We analyzed a total of 94 annotated genes [38-42] (see Additional file 8) for which we could retrieve the localization co-ordinates using Biomart. This analysis showed that the *Drosophila *mag genes would not generate any cluster when processed using *Gepoclu*. On the contrary, testes-specific genes in *D. melanogaster *are known to cluster together [36] but their homologues (K Magnusson, unpublished but see Additional files 7, 3 and 4) in *A. gambiae *are rather dispersed along the chromosome (according to our findings as detailed in the next example application), thus suggesting that mag and testis specific genes are under different selection pressure in *Anopheles *and *Drosophila*, possibly as consequence of monogamous versus polygamous mating behavior respectively. We also investigated mag clustering behavior by exploiting the functionality of *Gepoclu *to create clusters through an iterative process, when new genes were added to existing clusters: genes would either form independent clusters or merge into existing ones. We started by merging the mag and ubiquitous classes [28] and then we sequentially added the other tissue-specific groups using different positional distance thresholds. At a threshold value of 21 kbp genes coming from different datasets (i.e. different tissues) formed a single large cluster on chromosome 3R (Figure 5.A). The contents of such cluster are visible in Figure 5.B.

#### Example application 3: clustering analysis of mosquito sex-biased genes at different developmental stages - microarray data

**Figure 6:**
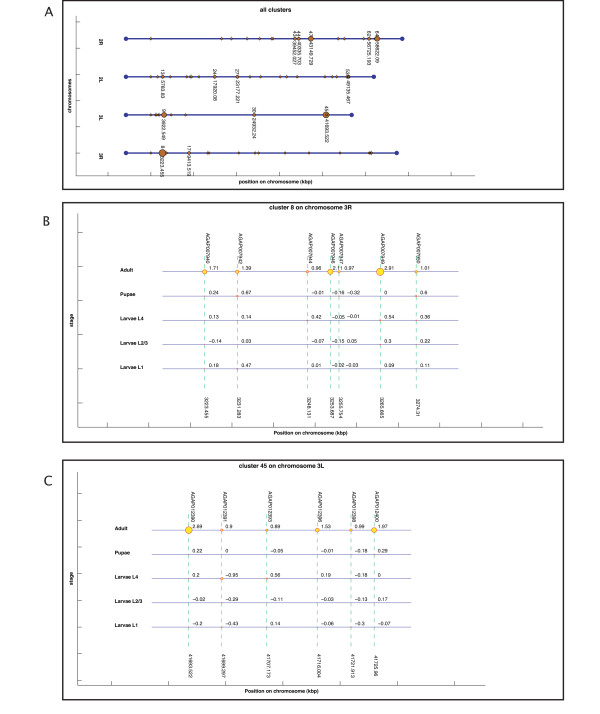
**Clustering results for male mosquitoes at different developmental stages**. Microarray data (see Additional data file 6 and 7). a) multiple-cluster view of the clustering results for male mosquitoes, only clusters containing more than two-genes were labeled; b,c) two example single-cluster views obtained from the cluster set. Multiple expression levels related to different developmental stages are visible.

In this analysis we investigated positional clustering of mosquito sex-biased genes from early development to adulthood. The extent and occurrence of gene positional clustering was analyzed at five different developmental stages: *larvae L1*, *larvae L2/3*, *larvae L4*, *pupae *and *adults *(see Additional file 7). Only genes uniquely expressed either in males or in females were considered; expression values for the five developmental stages were available as microarray data (see Additional file 3), while the list of testis-specific genes was found by RT-PCR (see Additional file 4). As control, a batch of statistical tests was run against clusters obtained from randomly selected genes as illustrated in the previous examples (see Additional file 7). The results showed that clusters obtained for the three larval female stages were not sufficiently differentiated from the random data sets; however, clusters obtained for male larvae, pupae and adults showed significant differentiation. Further analyses indicated that even at the adult stages, clustering tendency is not strongly differentiated between males and females. As an example of the clusters found, in Figure 6.A a multiple-cluster view of the clusters that were found for male mosquitoes is illustrated. Some clusters were also visualized by means of the single-cluster view functionality of *Gepoclu *(see Figure 6.B and Figure 6.C). In particular the single-cluster views are useful features as they allow for studying the progression of the expression values in the various developmental stages, for the genes contained in the cluster. The analysis also uncovered that the adult testes-specific genes did not form clusters (see Additional file 7), thus showing a different behavior with respect to the *Drosophila *ones (see example application 2).

## Conclusions

Gene positional clustering analysis is widely recognized as a fundamental tool towards a deeper comprehension of the mechanisms that lie underneath gene transcription regulation; widespread methodologies and bioinformatics tools of general applicability are increasingly needed to support this type of analysis. The problem is of growing importance, as an increasingly larger amount of gene-related information data is being accumulated thanks for large-scale gene analysis techniques such as microarray, EST and PCR. In particular, the investigation of positional clustering in conjunction with gene expression values seems promising towards the identification of hot spots of co-expressed and co-localized genes.

We have developed a software tool *Gepoclu *(Gene Positional Clustering) that automatically selects genes based on expression values from multiple sources, including microarray, EST and qRT-PCR, and performs positional clustering. *Gepoclu *provides expression-based gene selection from multiple experimental sources, position-based gene clustering and cluster visualization functionalities, all as parts of the same fully integrated, and interactive, package. This means rapid iterations while exploring for emergent behavior, and full programmability of the filtering and clustering steps.

*Gepoclu *is a useful data mining tool for exploring relationships among transcriptional data deriving form different sources. It can be operated by non-programmers with ease, by means of the suite of provided interactive applications, which can be run from the Matlab command line. Moreover, the whole set of functionalities provided by Gepoclu is fully programmable and can be configured to support specific application needs as well as novel analysis scenarios.

Three transcription data sets have been processed using *Gepoclu *to illustrate how the functional capabilities of this software may bring novel and useful results to the table, possibly by highlighting previously hidden relationships between data. The results of this analysis highlight both the advantages and the versatility of performing positional clustering with *Gepoclu*. The software disclosed hidden differences in the distribution of *Anopheles *and *Drosophila *sex biased genes and allowed the identification of a very large transcriptional hot-spot (on chromosome 3R), which could be targeted in the attempt to control mating behavior in *Anopheles*. The analysis of gene expression data in conjunction with co-localization information, as deduced by the proposed methodology introduces a paradigm shift, from more traditional approaches aimed at analyzing a single gene and its role in complex transcriptional phenomena.

## Availability and requirements

**• Project name: ***Gepoclu *(Gene positional clustering)

**• Project home page: **http://gepoclu.sourceforge.net/

**• Operating system(s): **Any operating system supporting Matlab

**• Programming language: **Matlab (release 2009b)

**• Other requirements: **None

**• Licenses: **no license required

## Abbreviations

bp: base pairs; EST: Expressed Sequence Tags;

## Authors' contributions

TD, NS and AC developed the concept. TD and NS wrote the software. TD and GM performed data analysis. KM provided the unpublished data set. TD and AC wrote the manuscript. All authors read and approved the final manuscript.

## Supplementary Material

Additional file 1 ***Gepoclu *flowcharts**. Detailed flowcharts documenting the entire analysis process supported by *Gepoclu*. Click here for file

Additional file 2 ***Gepoclu *software**. Matlab source code of the *Gepoclu *software. Click here for file

Additional file 3 **Microarray expressions for example application 3**. Excel spreadsheet containing the microarray expression data for example application 3. Click here for file

Additional file 4 **Testis-specific genes for example application 3**. Excel spreadsheet containing the list of testis-specific genes as found by RT-PCR for example application 3. Click here for file

Additional file 5 **Results for example application 1**. Tables reporting clustering results and their statistical significance for example application 1. Click here for file

Additional file 6 **Results for example application 2**. Tables reporting clustering results and their statistical significance for example application 2. Click here for file

Additional file 7 **Results for example application 3**. Tables reporting clustering results and their statistical significance for example application 3. Click here for file

Additional file 8 ***Drosophila *Acp genes for example application 2**. Table listing the *Drosophila *Acp genes used in the example application 2. Click here for file
