# Supplementary material for: Gepoclu: a software tool for identifying and analyzing gene positional clusters in large-scale gene expression analysis
Source: BMC Bioinformatics. 2011 Jan 26;12:34. doi: 10.1186/1471-2105-12-34 (PMC3040130; doi:10.1186/1471-2105-12-34)

Additional file 1

Flowcharts documenting the entire analysis process supported by *Gepoclu*.

(a) Gather gene information

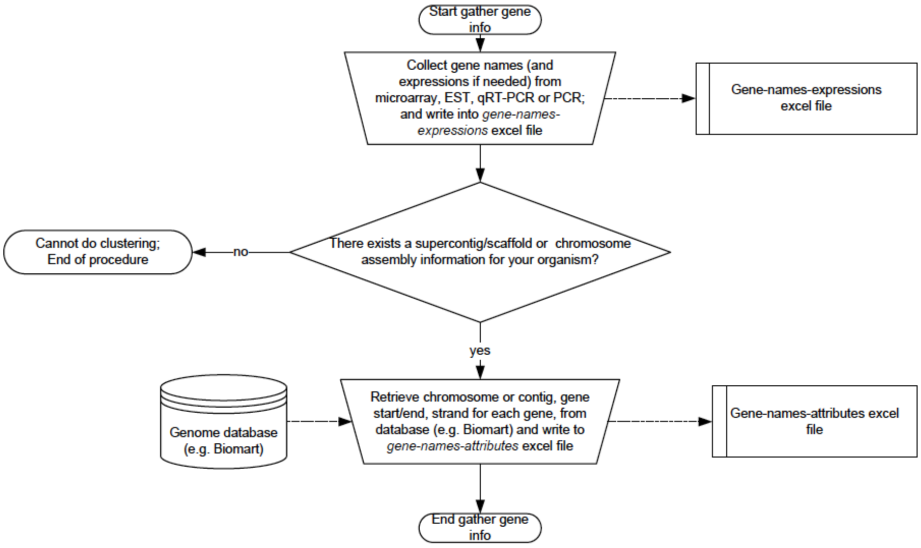

(b) Merge positional and expression info

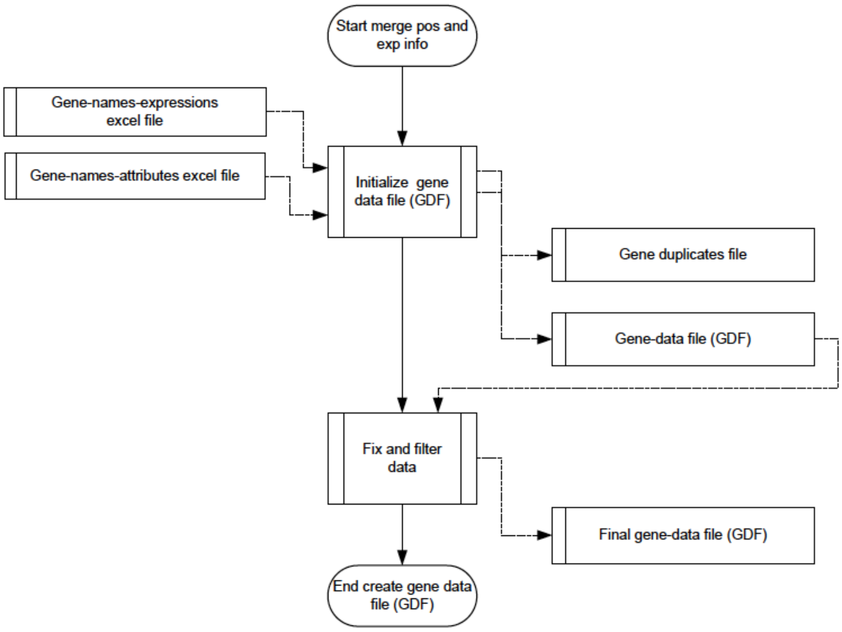

(c) Initialize Gene Data File (GDF)

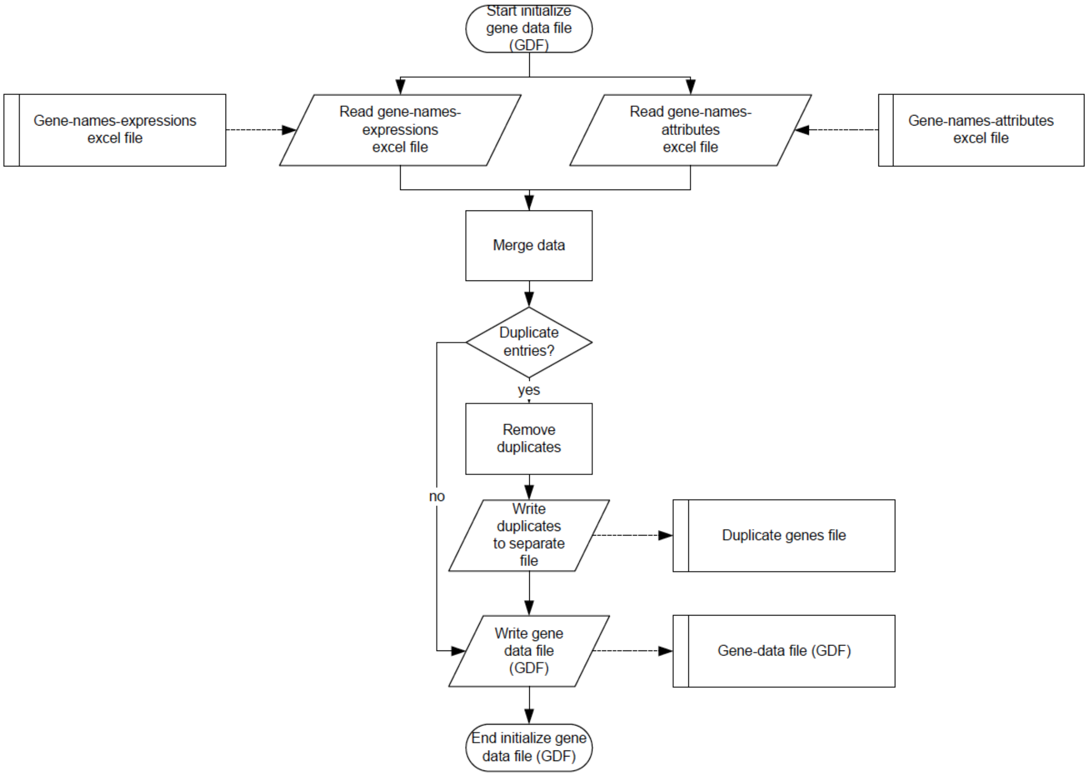

(d) Fix and Filter Data

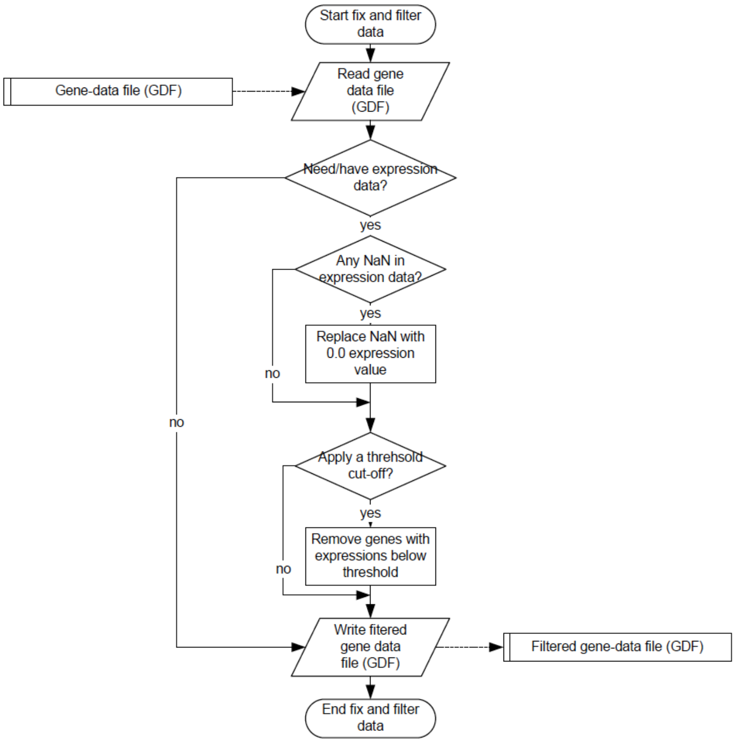

(e) Create Clusters

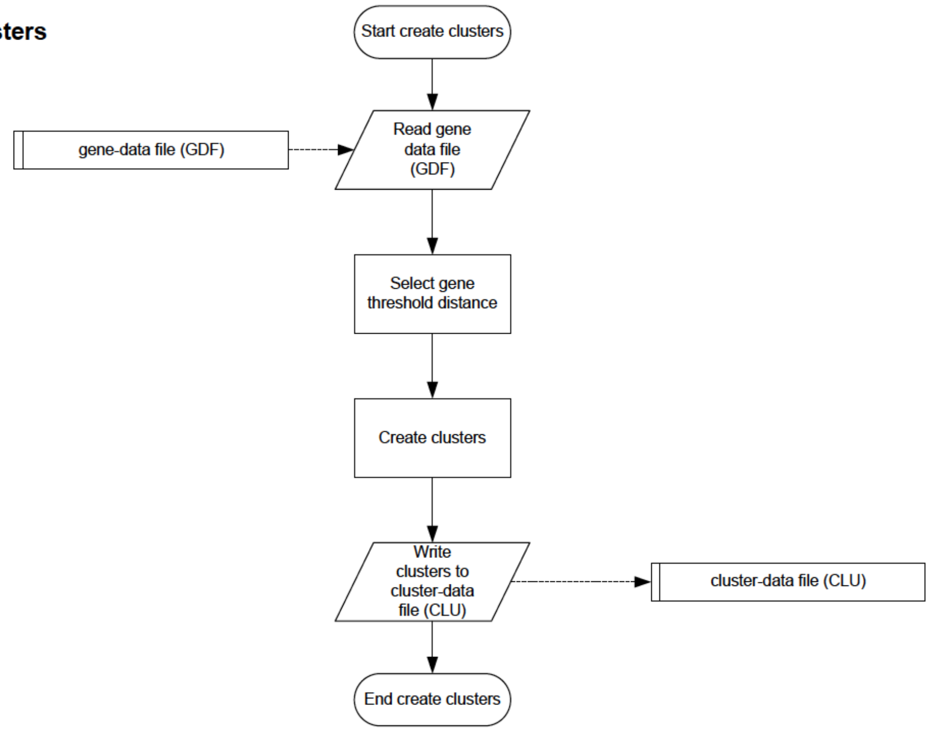

(f) Update clusters with new genes

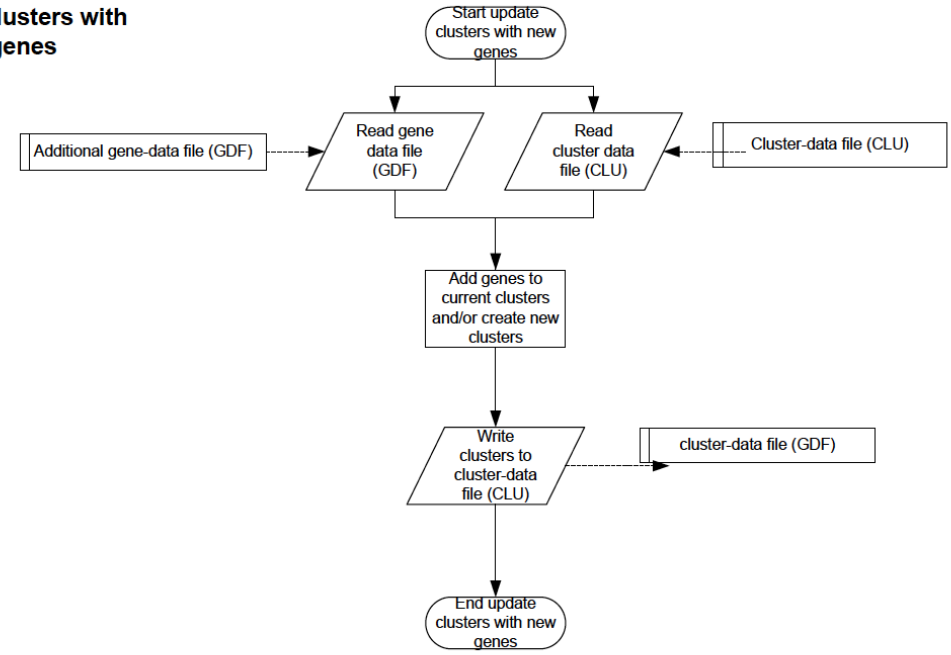

(g) Assessment of statistical significance of clustering

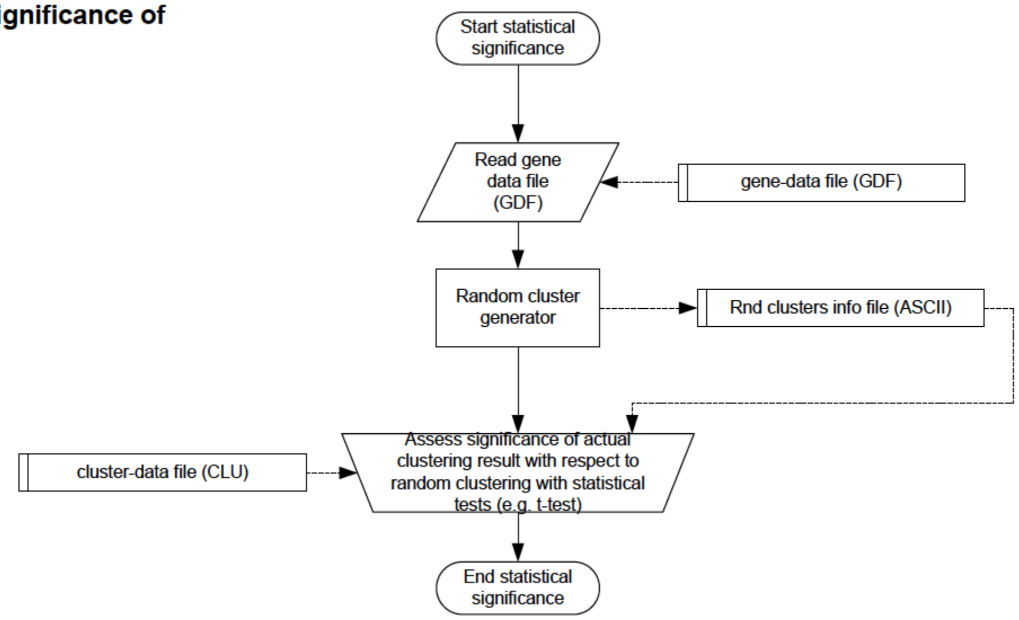

Supplement: Additional file 1 — Gepoclu flowcharts. Detailed flowcharts documenting the entire analysis process supported by Gepoclu. [file 1471-2105-12-34-S1.PDF]
