# Supplementary material for: Gepoclu: a software tool for identifying and analyzing gene positional clusters in large-scale gene expression analysis
Source: BMC Bioinformatics. 2011 Jan 26;12:34. doi: 10.1186/1471-2105-12-34 (PMC3040130; doi:10.1186/1471-2105-12-34)
Supplement: Additional file 5 — Results for example application 1. Tables reporting clustering results and their statistical significance for example application 1. [file 1471-2105-12-34-S5.PDF]

Additional File 5

Detailed clustering results and statistical significance assessment for example application 1

1. Clustering results on each gene dataset

SOURCE	N. of genes in the dataset	N. of genes after duplicate removal	N. of computed clusters	N. of clustered genes	% of clustered genes
NBF-3 up	2210	1883	0	0	0.0%
NBF-3 down	2333	1835	335	856	46.6%
3-24 up	4514	3667	823	2609	71.1%
3-24 down	2672	2179	397	1123	51.5%
24-48 up	1712	1422	247	589	41.4%
24-48 down	1496	1221	211	512	41.9%
48-72 up	604	542	65	145	26.8%
48-72 down	690	528	51	133	25.2%
72-96 up	1706	1340	252	619	46.2%
72-96 down	2185	1824	387	1014	55.6%
96-15 up	2	2	0	0	0.0%
96-15 down	211	179	16	42	23.5%

2. Clustering results on each gene dataset, compared with average clustering results on 20 datasets of randomly selected genes (statistical significance assessment).

Analyzed File/Set	N. of genes in clusters computed from the real dataset	N. of genes in clusters computed from the random dataset (avg.) [c]	One sample T test- t value d	One sample T test Sig. (2-tailed) [e]	95% Confidence Interval of the Difference (Lower\Upper) [f]	Conclusion [g]
NBF-3 up	0	761.9	188.383	0	753.43 \770.37	rnd>obs
NBF-3 down	856	743.55	-20.433	0	-123.97 \ -100.93	obs>rnd
3-24 up	2609	2200.45	-76.139	0	-419.78 \ -397.32	obs>rnd
3-24 down	1123	978.45	-21.094	0	-158.89 \ -130.21	obs>rnd
24-48 up	589	475.75	-29.845	0	-121.19 \ -105.31	obs>rnd
24-48 down	512	365.7	-31.024	0	-156.17 \ -136.43	obs>rnd
48-72 up	145	81.55	-21.135	0	-69.73 \ -57.17	obs>rnd
48-72 down	133	80	-24.447	0	-57.54 \ -48.46	obs>rnd
72-96 up	619	438.25	-30.705	0	-193.07 \ -168.43	obs>rnd
72-96 down	1014	724.75	-69.083	0	-298.01 \ -280.49	obs>rnd
96-15 up	0	0	NA	NA	NA	NA
96-15 down	42	9.3	-37.291	0	-34.54 \ -30.86	obs>rnd

[c] At each run , Gepoclu randomly extracted genes to form a random dataset the same size of the actual dataset, and did the clustering analysis on such set. The clustering results were averaged over 20 runs. The random gene selection was done on 12457 *Anopheles* genes as retrieved by Biomart.

[d,e,f] One-sample Student's t-test statistics obtained with SPSS.

[g] obs>rnd: the n. of clusters computed from the real dataset (observed) is larger than the average number of clusters computed from the random (rnd) datasets; rnd>obs: the opposite is true.

3. Additional analyses on the clustering results on each gene dataset

	Cluster size	N. of clusters	N. of genes in clusters of such size
NBF-3 up	2	0	0
	≥3	0	0
	Tot	0	0

	Cluster size	N. of clusters	N. of genes in clusters of such size
NBF-3 down	2	228	456
	3	69	207
	4	19	76
	5	8	40
	6	7	42
	7	2	14
	10	1	10
	11	1	11
	≥12	0	0
	Tot	335	856

	Cluster size	N. of clusters	N. of genes in clusters of such size
3-24 up	2	389	778
	3	205	615
	4	113	452
	5	45	225
	6	28	168
	7	13	91
	8	19	152
	9	3	27
	10	3	30
	11	1	11
	12	1	12
	15	1	15
	16	1	16
	17	1	17
	≥18	0	0
	Tot	823	2609

3-24 down	Cluster size	N. of clusters	N. of genes in clusters of such size
	2	233	466
	3	97	291
	4	33	132
	5	17	85
	6	6	36
	7	3	21
	8	3	24
	10	1	10
	11	3	33
	25	1	25
	≥26	0	0
	Tot	397	1123

24-48 up	Cluster size	N. of clusters	N. of genes in clusters of such size
	2	182	364
	3	49	147
	4	10	40
	5	1	5
	6	2	12
	7	3	21
	≥8	0	0
	Tot	247	589

24-48 down	Cluster size	N. of clusters	N. of genes in clusters of such size
	2	148	296
	3	45	135
	4	11	44
	5	5	25
	6	2	12
	≥7	0	0
	Tot	211	512

48-72 up	Cluster size	N. of clusters	N. of genes in clusters of such size
	2	53	106
	3	10	30
	4	1	4
	5	1	5
	≥6	0	0
	Tot	65	145

	Cluster size	N. of clusters	N. of genes in clusters of such size
48-72 down	2	38	76
	3	4	12
	4	5	20
	5	1	5
	6	2	12
	8	1	8
	≥7	0	0
	Tot	51	133

	Cluster size	N. of clusters	N. of genes in clusters of such size
72-96 up	2	183	366
	3	43	129
	4	16	64
	5	6	30
	6	2	12
	8	1	8
	10	1	10
	≥11	0	0
	Tot	252	619

	Cluster size	N. of clusters	N. of genes in clusters of such size
72-96 down	2	243	486
	3	98	294
	4	23	92
	5	10	50
	6	6	36
	7	3	21
	8	1	8
	9	3	27
	≥11	0	0
	Tot	387	1014

	Cluster size	N. of clusters	N. of genes in clusters of such size
96-15 up	2	0	0
	3	0	0
	4	0	0
	≥6	0	0
	Tot	0	0

96-15 down	Cluster size	N. of clusters	N. of genes in clusters of such size
	2	10	20
	3	4	12
	4	1	4
	6	1	6
	≥7	0	0
	Tot	16	42

4. Chromosome Bias Distribution

4.1. Analysis of specific dataset "3h up".

Clustering results for the specific dataset "3h up", compared with a) clustering results computed on the entire *A. gambiae* genome of 13683 genes as retrieved from (Holt et al, 2002); b) clustering results computed on the protein-coding genes of *A. gambiae* (12457 genes, as obtained from Biomart).

3h up	N. of genes of the specific dataset, in clusters with size ≥3	% of genes of the specific dataset, in clusters with size≥3	% of genes of the complete dataset (Holt et al. 2002), in clusters with size ≥3	Ratio of specific dataset / complete dataset	% of genes of the protein-coding dataset (Biomart), in clusters with size ≥3	Ratio of specific dataset / Biomart dataset
X	0	0	8.4%	0	8.7%	0
2L	0	0	24.7%	0	23.3%	0
2R	0	0	29.6%	0	27.6%	0
3L	0	0	16.1%	0	16.3%	0
3R	0	0	20.1%	0	20.1%	0
UNKN	0	0	1.2%	0	3.9%	0
Total	0					

Specific dataset: "3h down".

3h down	N. of genes of the specific dataset, in clusters with size ≥3	% of genes of the specific dataset, in clusters with size≥3	% of genes of the complete dataset (Holt et al. 2002), in clusters with size ≥3	Ratio of specific dataset / complete dataset	% of genes of the protein-coding dataset (Biomart), in clusters with size ≥3	Ratio of specific dataset / Biomart dataset
X	36	9.0%	8.4%	1.07	8.7%	1.03
2L	106	26.5%	24.7%	1.07	23.3%	1.14
2R	115	28.8%	29.6%	0.97	27.6%	1.04
3L	52	13.0%	16.1%	0.81	16.3%	0.80
3R	91	22.8%	20.1%	1.13	20.1%	1.13
UNKN	0	0.0%	1.2%	0.00	3.9%	0.00
**Total**	400					

Specific dataset: "3-24 up".

3-24 up	N. of genes of the specific dataset, in clusters with size ≥3	% of genes of the specific dataset, in clusters with size≥3	% of genes of the complete dataset (Holt et al. 2002), in clusters with size ≥3	Ratio of specific dataset / complete dataset	% of genes of the protein-coding dataset (Biomart), in clusters with size ≥3	Ratio of specific dataset / Biomart dataset
X	74	4.0%	8.4%	0.48	8.7%	0.46
2L	447	24.4%	24.7%	0.99	23.3%	1.05
2R	621	33.9%	29.6%	1.15	27.6%	1.23
3L	299	16.3%	16.1%	1.02	16.3%	1.00
3R	384	21.0%	20.1%	1.05	20.1%	1.04
UNKN	6	0.3%	1.2%	0.28	3.9%	0.08
Total	1831					

Specific dataset: "3-24 down".

3-24 down	N. of genes of the specific dataset, in clusters with size ≥3	% of genes of the specific dataset, in clusters with size≥3	% of genes of the complete dataset (Holt et al. 2002), in clusters with size ≥3	Ratio of specific dataset / complete dataset	% of genes of the protein-coding dataset (Biomart), in clusters with size ≥3	Ratio of specific dataset / Biomart dataset
X	25	3.8%	8.4%	0.45	8.7%	0.44
2L	218	33.2%	24.7%	1.34	23.3%	1.42
2R	180	27.4%	29.6%	0.93	27.6%	0.99
3L	95	14.5%	16.1%	0.90	16.3%	0.89
3R	133	20.2%	20.1%	1.01	20.1%	1.01
UNKN	6	0.9%	1.2%	0.78	3.9%	0.23
Total	657					

Specific dataset: "24-48 up".

24-48 up	N. of genes of the specific dataset, in clusters with size ≥3	% of genes of the specific dataset, in clusters with size≥3	% of genes of the complete dataset (Holt et al. 2002), in clusters with size ≥3	Ratio of specific dataset / complete dataset	% of genes of the protein-coding dataset (Biomart), in clusters with size ≥3	Ratio of specific dataset / Biomart dataset
X	21	9.3%	8.4%	1.11	8.7%	1.07
2L	56	24.9%	24.7%	1.01	23.3%	1.07
2R	61	27.1%	29.6%	0.92	27.6%	0.98
3L	49	21.8%	16.1%	1.36	16.3%	1.34
3R	38	16.9%	20.1%	0.84	20.1%	0.84
UNKN	0	0.0%	1.2%	0.00	3.9%	0.00
Total	225					

Specific dataset: "24-48 down".

24-48 down	N. of genes of the specific dataset, in clusters with size ≥3	% of genes of the specific dataset, in clusters with size≥3	% of genes of the complete dataset (Holt et al. 2002), in clusters with size ≥3	Ratio of specific dataset / complete dataset	% of genes of the protein-coding dataset (Biomart), in clusters with size ≥3	Ratio of specific dataset / Biomart dataset
X	19	8.8%	8.4%	1.04	8.7%	1.01
2L	68	31.5%	24.7%	1.27	23.3%	1.35
2R	58	26.9%	29.6%	0.91	27.6%	0.97
3L	19	8.8%	16.1%	0.55	16.3%	0.54
3R	49	22.7%	20.1%	1.13	20.1%	1.13
UNKN	3	1.4%	1.2%	1.19	3.9%	0.35
Total	216					

Specific dataset: "48-72 up".

48-72 up	N. of genes of the specific dataset, in clusters with size ≥3	% of genes of the specific dataset, in clusters with size≥3	% of genes of the complete dataset (Holt et al. 2002), in clusters with size ≥3	Ratio of specific dataset / complete dataset	% of genes of the protein-coding dataset (Biomart), in clusters with size ≥3	Ratio of specific dataset / Biomart dataset
X	0	0.0%	8.4%	0.00	8.7%	0.00
2L	16	41.0%	24.7%	1.66	23.3%	1.76
2R	6	15.4%	29.6%	0.52	27.6%	0.56
3L	6	15.4%	16.1%	0.96	16.3%	0.95
3R	11	28.2%	20.1%	1.41	20.1%	1.40
UNKN	0	0.0%	1.2%	0.00	3.9%	0.00
Total	39					

Specific dataset: "48-72 down".

48-72 down	N. of genes of the specific dataset, in clusters with size ≥3	% of genes of the specific dataset, in clusters with size≥3	% of genes of the complete dataset (Holt et al. 2002), in clusters with size ≥3	Ratio of specific dataset / complete dataset	% of genes of the protein-coding dataset (Biomart), in clusters with size ≥3	Ratio of specific dataset / Biomart dataset
X	10	17.5%	8.4%	2.08	8.7%	2.01
2L	15	26.3%	24.7%	1.07	23.3%	1.13
2R	14	24.6%	29.6%	0.83	27.6%	0.89
3L	10	17.5%	16.1%	1.09	16.3%	1.08
3R	8	14.0%	20.1%	0.70	20.1%	0.70
UNKN	0	0.0%	1.2%	0.00	3.9%	0.00
Total	57					

Specific dataset: “72-96 up”.

72-96 up	N. of genes of the specific dataset, in clusters with size ≥3	% of genes of the specific dataset, in clusters with size≥3	% of genes of the complete dataset (Holt et al. 2002), in clusters with size ≥3	Ratio of specific dataset / complete dataset	% of genes of the protein-coding dataset (Biomart), in clusters with size ≥3	Ratio of specific dataset / Biomart dataset
X	14	5.5%	8.4%	0.66	8.7%	0.63
2L	69	27.3%	24.7%	1.10	23.3%	1.17
2R	72	28.5%	29.6%	0.96	27.6%	1.03
3L	48	19.0%	16.1%	1.18	16.3%	1.17
3R	50	19.8%	20.1%	0.99	20.1%	0.98
UNKN	0	0.0%	1.2%	0.00	3.9%	0.00
Total	253					

Specific dataset: “72-96 down”

72-96 down	N. of genes of the specific dataset, in clusters with size ≥3	% of genes of the specific dataset, in clusters with size≥3	% of genes of the complete dataset (Holt et al. 2002), in clusters with size ≥3	Ratio of specific dataset / complete dataset	% of genes of the protein-coding dataset (Biomart), in clusters with size ≥3	Ratio of specific dataset / Biomart dataset
X	10	1.9%	8.4%	0.22	8.7%	0.22
2L	146	27.7%	24.7%	1.12	23.3%	1.19
2R	184	34.8%	29.6%	1.18	27.6%	1.26
3L	97	18.4%	16.1%	1.14	16.3%	1.13
3R	88	16.7%	20.1%	0.83	20.1%	0.83
UNKN	3	0.6%	1.2%	0.48	3.9%	0.14
Total	528					

Specific dataset: “96-15 up”

96-15 up	N. of genes of the specific dataset, in clusters with size ≥3	% of genes of the specific dataset, in clusters with size≥3	% of genes of the complete dataset (Holt et al. 2002), in clusters with size ≥3	Ratio of specific dataset / complete dataset	% of genes of the protein-coding dataset (Biomart), in clusters with size ≥3	Ratio of specific dataset / Biomart dataset
X	0	0	8.4%	0	8.7%	0
2L	0	0	24.7%	0	23.3%	0
2R	0	0	29.6%	0	27.6%	0
3L	0	0	16.1%	0	16.3%	0
3R	0	0	20.1%	0	20.1%	0
UNKN	0	0	1.2%	0	3.9%	0
Total	0					

Specific dataset: “96-15 down”

96-15 down	N. of genes of the specific dataset, in clusters with size ≥3	% of genes of the specific dataset, in clusters with size≥3	% of genes of the complete dataset (Holt et al. 2002), in clusters with size ≥3	Ratio of specific dataset / complete dataset	% of genes of the protein-coding dataset (Biomart), in clusters with size ≥3	Ratio of specific dataset / Biomart dataset
X	3	13.6%	8.4%	1.62	8.7%	1.56
2L	0	0.0%	24.7%	0.00	23.3%	0.00
2R	10	45.5%	29.6%	1.54	27.6%	1.64
3L	3	13.6%	16.1%	0.85	16.3%	0.84
3R	6	27.3%	20.1%	1.36	20.1%	1.36
UNKN	0	0.0%	1.2%	0.00	3.9%	0.00
Total	22					
