# Supplementary material for: Gepoclu: a software tool for identifying and analyzing gene positional clusters in large-scale gene expression analysis
Source: BMC Bioinformatics. 2011 Jan 26;12:34. doi: 10.1186/1471-2105-12-34 (PMC3040130; doi:10.1186/1471-2105-12-34)
Supplement: Additional file 6 — Results for example application 2. Tables reporting clustering results and their statistical significance for example application 2. [file 1471-2105-12-34-S6.PDF]

Additional File 6

Detailed clustering results and statistical significance assessment for example application 2

1. Clustering results on each gene dataset

SOURCE	N. of genes in the dataset	N. of genes after duplicate removal	N. of computed clusters	N. of clustered genes	% of clustered genes
set1_MAG	19	19	4	14	73.7%
set2_MAG_T	10	10	1	7	70.0%
set3_MAG_T_F	6	6	1	2	33.3%
set4_MAG_RB_F	4	4	1	2	50.0%
set5_UBIQUITOUS	11	11	0	0	0.0%

2. Clustering results on each gene dataset, compared with average clustering results on 20 datasets of randomly selected genes (statistical significance assessment).

Analyzed File/Set	N. of genes in clusters computed from the real dataset	N. of genes in clusters computed from the random dataset (avg.) [c]	One sample T test- t value d	One sample T test Sig. (2-tailed) [e]	95% Confidence Interval of the Difference (Lower\Upper) [f]	Conclusion [g]
set1_MAG	14	0	NA	NA	NA	obs>rnd
set2_MAG_T	7	0	NA	NA	NA	obs>rnd
set3_MAG_T_F	2	0	NA	NA	NA	obs>rnd
set4_MAG_RB_F	2	0	NA	NA	NA	obs>rnd
set5_UBIQUITOUS	0	0	NA	NA	NA	No difference

[c] At each run , Gepoclu randomly extracted genes to form a random dataset the same size of the actual dataset, and did the clustering analysis on such set. The clustering results were averaged over 20 runs. The random gene selection was done on 12457 *Anopheles* genes as retrieved by Biomart.

[d,e,f] One-sample Student's t-test statistics obtained with SPSS.

[g] obs>rnd: the n. of clusters computed from the real dataset (observed) is larger than the average number of clusters computed from the random (rnd) datasets; rnd>obs: the opposite is true.

3. Additional analyses on the clustering results on gene dataset set1_MAG

	Cluster size	N. of clusters	N. of genes in clusters of such size
set1_MAG	2	2	4
	3	1	3
	7	1	7
	≥8	0	0
	Tot	4	14

4. Chromosome Bias Distribution

4.1. Analysis of specific dataset "set1_MAG".

Clustering results for the specific dataset "set1_MAG", compared with a) clustering results computed on the entire *A. gambiae* genome of 13683 genes as retrieved from (Holt et al, 2002); b) clustering results computed on the protein-coding genes of *A. gambiae* (12457 genes, as obtained from Biomart).

set1_MAG	N. of genes of the specific dataset, in clusters with size ≥2	% of genes of the specific dataset, in clusters with size≥2	% of genes of the complete dataset (Holt et al. 2002), in clusters with size ≥2	Ratio of specific dataset / complete dataset	% of genes of the protein-coding dataset (Biomart), in clusters with size ≥2s	Ratio of specific dataset / Biomart dataset
X	0	0.0%	8.4%	0.00	8.7%	0.00
2L	2	14.3%	20.1%	0.71	23.3%	0.61
2R	0	0.0%	16.1%	0.00	27.6%	0.00
3L	0	0.0%	29.6%	0.00	16.3%	0.00
3R	10	71.4%	24.7%	2.89	20.1%	3.55
UNKN	2	14.3%	1.2%	12.19	3.9%	3.63
Total	14					

Specific dataset: "set2_MAG_T".

set2_MAG_T	N. of genes of the specific dataset, in clusters with size ≥2	% of genes of the specific dataset, in clusters with size≥2	% of genes of the complete dataset (Holt et al. 2002), in clusters with size ≥2	Ratio of specific dataset / complete dataset	% of genes of the protein-coding dataset (Biomart), in clusters with size ≥2	Ratio of specific dataset / Biomart dataset
X	0	0.0%	8.4%	0.00	8.7%	0.00
2L	0	0.0%	20.1%	0.00	23.3%	0.00
2R	0	0.0%	16.1%	0.00	27.6%	0.00
3L	0	0.0%	29.6%	0.00	16.3%	0.00
3R	7	100.0%	24.7%	4.05	20.1%	4.97
UNKN	0	0.0%	1.2%	0.00	3.9%	0.00
Total	7					

Specific dataset: “set3_MAG_T_F”.

Set3_MAG_T_F	N. of genes of the specific dataset, in clusters with size ≥2	% of genes of the specific dataset, in clusters with size≥2	% of genes of the complete dataset (Holt et al. 2002), in clusters with size ≥2	Ratio of specific dataset / complete dataset	% of genes of the protein-coding dataset (Biomart), in clusters with size ≥2	Ratio of specific dataset / Biomart dataset
X	0	0.0%	8.4%	0.00	8.7%	0.00
2L	2	100.0%	20.1%	4.99	23.3%	4.29
2R	0	0.0%	16.1%	0.00	27.6%	0.00
3L	0	0.0%	29.6%	0.00	16.3%	0.00
3R	0	0.0%	24.7%	0.00	20.1%	0.00
UNKN	0	0.0%	1.2%	0.00	3.9%	0.00
Total	2					

Specific dataset: “set4_MAG_RB_F”.

set4_MAG_RB_F	N. of genes of the specific dataset, in clusters with size ≥2	% of genes of the specific dataset, in clusters with size≥2	% of genes of the complete dataset (Holt et al. 2002), in clusters with size ≥2	Ratio of specific dataset / complete dataset	% of genes of the protein-coding dataset (Biomart), in clusters with size ≥2	Ratio of specific dataset / Biomart dataset
X	0	0.0%	8.4%	0.00	8.7%	0.00
2L	2	100.0%	20.1%	4.99	23.3%	4.29
2R	0	0.0%	16.1%	0.00	27.6%	0.00
3L	0	0.0%	29.6%	0.00	16.3%	0.00
3R	0	0.0%	24.7%	0.00	20.1%	0.00
UNKN	0	0.0%	1.2%	0.00	3.9%	0.00
Total	2					

Specific dataset: “set5_UBIQUITOUS”.

set5_UBIQUITOUS	N. of genes of the specific dataset, in clusters with size ≥2	% of genes of the specific dataset, in clusters with size≥2	% of genes of the complete dataset (Holt et al. 2002), in clusters with size ≥2	Ratio of specific dataset / complete dataset	% of genes of the protein-coding dataset (Biomart), in clusters with size ≥2	Ratio of specific dataset / Biomart dataset
X	0	0	8.4%	NA	8.7%	NA
2L	0	0	20.1%	NA	23.3%	NA
2R	0	0	16.1%	NA	27.6%	NA
3L	0	0	29.6%	NA	16.3%	NA
3R	0	0	24.7%	NA	20.1%	NA
UNKN	0	0	1.2%	NA	3.9%	NA
Total	0					
