# Supplementary material for: Gepoclu: a software tool for identifying and analyzing gene positional clusters in large-scale gene expression analysis
Source: BMC Bioinformatics. 2011 Jan 26;12:34. doi: 10.1186/1471-2105-12-34 (PMC3040130; doi:10.1186/1471-2105-12-34)
Supplement: Additional file 7 — Results for example application 3. Tables reporting clustering results and their statistical significance for example application 3. [file 1471-2105-12-34-S7.PDF]

## Additional File 7

Detailed clustering results and statistical significance assessment for example application 3

### 1. Clustering results on each gene dataset

| SOURCE | N. of genes in the dataset | N. of genes after duplicate removal | N. of computed clusters | N. of clustered genes | % of clustered genes |
|---|---|---|---|---|---|
| Larvae Male | 144 | 144 | 5 | 11 | 7.6% |
| Larvae Female | 32 | 32 | 0 | 0 | 0 |
| Pupae Male | 65 | 65 | 1 | 2 | 3.0% |
| Pupae Female | 45 | 45 | 1 | 2 | 3.0% |
| Adult Male | 617 | 617 | 64 | 156 | 25.2% |
| Adult Female | 1113 | 1113 | 198 | 483 | 43.3% |
| Testes | 97 | 97 | 1 | 2 | 2.1% |

### 2. Clustering results on each gene dataset, compared with average clustering results on 20 datasets of randomly selected genes (statistical significance assessment)

| Analyzed File/Set | N. of genes in clusters computed from the real dataset | N. of genes in clusters computed from the random dataset (avg.) [c] | One sample T test- t value d | One sample T test Sig. (2-tailed) [e] | 95% Confidence Interval of the Difference (Lower\Upper) [f] | Conclusion [g] |
|---|---|---|---|---|---|---|
| Larvae Male | 11 | 6.80 | -6.370 | 0 | -5.58 \ -2.82 | obs>rnd |
| Larvae Female | 0 | 0.2 | 1.453 | 0.163 | -0.09 \ 0.49 | No difference |
| Pupae Male | 2 | 0.8 | -4.485 | 0 | -1.76 \ -0.64 | obs>rnd |
| Pupae Female | 2 | 0.4 | -8.718 | 0 | -1.98 \ -1.22 | obs>rnd |
| Adult Male | 156 | 111.3 | -16.530 | 0 | -50.30 \ -39.00 | obs>rnd |
| Adult Female | 483 | 312.25 | -42.410 | 0 | -179.18 \ -162.32 | obs>rnd |
| Testes | 2 | 2.9 | 1.917 | 0.07 | -0.08 \ 1.88 | No difference |

[c] At each run , Gepoclu randomly extracted genes to form a random dataset the same size of the actual dataset, and did the clustering analysis on such set. The clustering results were averaged over 20 runs. The random gene selection was done on 12457 *Anopheles* genes as retrieved by Biomart.

[d,e,f] One-sample Student's t-test statistics obtained with SPSS.

[g] obs>rnd: the n. of clusters computed from the real dataset (observed) is larger than the average number of clusters computed from the random (rnd) datasets; rnd>obs: the opposite is true.
