# Supplementary material for: Gepoclu: a software tool for identifying and analyzing gene positional clusters in large-scale gene expression analysis
Source: BMC Bioinformatics. 2011 Jan 26;12:34. doi: 10.1186/1471-2105-12-34 (PMC3040130; doi:10.1186/1471-2105-12-34)
Supplement: Additional file 8 — Drosophila Acp genes for example application 2. Table listing the Drosophila Acp genes used in the example application 2. [file 1471-2105-12-34-S8.PDF]

Additional File 8

Drosophila Acp genes

GENEID	Chromosome
FBgn0041180	2L
FBgn0034638	2R
FBgn0015585	3L
FBgn0034195	2R
FBgn0038067	3R
FBgn0040341	X
FBgn0031260	2L
FBgn0033550	2R
FBgn0020509	3L
FBgn0035933	3L
FBgn0040376	X
FBgn0035165	3L
FBgn0250847	2L
FBgn0038419	3R
FBgn0020309	2L
FBgn0015287	3L
FBgn0033448	2R
FBgn0045823	3L
FBgn0032275	2L
FBgn0039043	3R
FBgn0032596	2L
FBgn0250842	2R
FBgn0037004	3L
FBgn0003034	3L
FBgn0015583	2L
FBgn0038919	3R
FBgn0002863	3R
FBgn0003512	3R
FBgn0036796	3L

**FBgn0043825**	**2L**
**FBgn0039623**	**3R**
**FBgn0037466**	**3R**
**FBgn0026262**	**4**
**FBgn0027280**	**X**
**FBgn0250874**	**X**
**FBgn0034753**	**2R**
**FBgn0023529**	**X**
**FBgn0021979**	**2R**
**FBgn0013745**	**3R**
**FBgn0051872**	**2L**
**FBgn0024211**	**3R**
**FBgn0015586**	**3L**
**FBgn0001218**	**X**
**FBgn0004926**	**3L**
**FBgn0029755**	**X**
**FBgn0032138**	**2L**
**FBgn0023415**	**2L**
**FBgn0034229**	**2R**
**FBgn0011670**	**3R**
**FBgn0011669**	**3R**
**FBgn0031908**	**2L**
**FBgn0038055**	**3R**
**FBgn0038950**	**3R**
**FBgn0038389**	**3R**
**FBgn0039562**	**3R**
**FBgn0034902**	**2R**
**FBgn0015790**	**3R**
**FBgn0036154**	**3L**
**FBgn0029157**	**3R**
**FBgn0036970**	**3L**
**FBgn0029688**	**X**

FBgn0030932	X
FBgn0032361	2L
FBgn0038918	3R
FBgn0032400	2L
FBgn0038985	3R
FBgn0014002	3L
FBgn0032521	2L
FBgn0027532	3L
FBgn0011559	2L
FBgn0036493	3L
FBgn0041094	3L
FBgn0031003	X
FBgn0028499	3R
FBgn0033999	2R
FBgn0028987	2L
FBgn0010406	3L
FBgn0027950	3R
FBgn0033029	2R
FBgn0033915	2R
FBgn0015584	2R
FBgn0002855	2L
FBgn0002856	2L
FBgn0031746	2L
FBgn0011668	3R
FBgn0035216	3L
FBgn0028986	2L
FBgn0002736	2R
FBgn0005585	3R
FBgn0037749	3R
FBgn0039787	3R
FBgn0010470	2R
FBgn0039591	3R

**FBgn0039597**	**3R**
