# Supplementary material for: Gepoclu: a software tool for identifying and analyzing gene positional clusters in large-scale gene expression analysis
Source: BMC Bioinformatics. 2011 Jan 26;12:34. doi: 10.1186/1471-2105-12-34 (PMC3040130; doi:10.1186/1471-2105-12-34)
Supplement: Additional file 2 — Gepoclu software. Matlab source code of the Gepoclu software. [file 1471-2105-12-34-S2.ZIP › Additional file 2/gepoclu v3.1.zip!/gepoclu v3.1/doc/instructions v3.1-eng.docx]

**Gepoclu – Gene Positional Clustering**

*Release: 3.1*

**Interactive functions documentation**

*This document describes the main interactive applications provided with Gepoclu. The applications work as the front-end of the entire Gepoclu library, they provide basic TTY interaction and are implemented as Matlab scripts which can be launched from the Matlab command line.*

*The application scripts are recognizable as they begin with the “i” letter.*

**============================**

**GDF creator	(matlab: iGdfCreator)**

**============================**

**SCHEMA**

**=========**

```
xls expressions ---->
                      iGdfCreator  ------------> geneDataFile
xls attributes ----->
```

**DESCRIPTION**

**==============**

This application merges the two Excel files containing gene positional and expression information. During merging, duplicate genes are identified (based on name) and removed. In duplicate removal, only the first encountered instance of a gene is kept. The resulting information is formatted as a text-based, gene data file (GDF); the names of the duplicate genes are written to a separate text file.

The two Excel files must be formatted so that they conform to the following requirements:

Expression file (.xls)

genename expr1 expr2 expr3 expr4 .....

genename expr1 expr2 expr3 expr4 .....

.......

Position file (.xls)

```
genename chrom  start end strand
genename chrom  start end strand
 ...
```

The two excel files must not contain any header or additional data.

If positional clustering is to be done regardless of gene expression data information, an expression file with expression values set to zero must be created. Names in the expression and position files must correspond to the same genes. The expression file supports multiple gene expressions, missing expression values are replaced by *NaN* (not a number) strings at merging. Expression values may be provided as ratios, normalized, logarithmic values, depending on the experiment (microarray, qRT-PCR, EST, PCR) or in any other user-defined format, as long as all the values are defined in the same reference so that they can be compared. The position file must contain all the required information for each gene: chromosome name, start and end in base pairs, and strand (encoded as ±1). The required format for the position file is consistent with the output format provided by the Biomart database. Merged gene information is written to a text file in the GDF (Gene data file) format. The format is defined as follows:

Gene data file (.gdf)

dset genename chrom start end strand expr1 expr2 expr3 expr4 .....

dset genename chrom start end strand expr1 expr2 expr3 expr4 .....

.....

Where the only additional information is a reference name for the dataset (*dset*, sometimes also called *origin*), provided by the user during the interactive merging session, and which is useful

when multiple GDF files must be used in iterative clustering investigation processes.

Genes listed in the GDF files are listed in properly sorted order, according to position on each chromosome.

**GUIDE TO OPERATION**

**====================**

The GDF creator is interactive. From the matlab prompt, type “iGdfCreator” (enter) and answer to the following questions:

*- name of the data set:*

this is the name of the experiment (also known as “origin”). Do not include blanks in the name.

*- name of excel file containing expressions:*

name of the excel file formatted to contain expression data.

*- name of the excel file containing attributes:*

name of the excel file properly formatted to contain the other attributes (chromosome, position, strand, etc.)

*- output gdf file:*

name of the text file that will contain the genes in GDF format.

*- duplicates file:*

name of the text file that will contain the names of the duplicate genes which have been removed during merging.

**================================**

**GDF NaN fixer	(iGdfNaNFilter)**

**================================**

**SCHEMA**

**========**

GeneDataFile -----------> iGdfNaNFixer ------------> GeneDataFile

**DESCRIPTION**

============

This application amends a GDF file by replacing *NaN* (Not a Number) expression values with a value provided by the user. The fixed file is saved as a new text file, in the GDF format.

Typical usage includes replacing NaN with 0.

**GUIDE TO OPERATION**

**====================**

From the Matlab prompt, type “"iGdfNaNFixer" (enter) and anwer the following questions:

*- gdf input file:*

name of the input file (in GDF format)

*- gdf output file:*

name of the output file (in GDF format)

*- replacement:*

numerical value to replace NaN with.

**===========================**

**GDF Filter	(matlab: iGdfFilter)**

**===========================**

**SCHEMA**

**========**

GeneDataFile -----------> iGdfFilter ------------> GeneDataFile

**DESCRIPTION**

**=============**

This application is used to discard/keep genes based on a threshold defined on expression values. The input is a GDF file and the output is another GDF file containing only the genes that passed the threshold criterion. The threshold can be applied to a single expression or to all expressions. The rule can be to keep genes above or below the threshold.

**GUIDE TO OPERATION**

**====================**

At the Matlab prompt, type “iGdfFilter” (enter) and aswer the following questions:

*- gdf input file:*

name of the input file (GDF format)

*- gdf output file:*

name of the output file (GDF format)

*- number of expression indices:*

since an array of indices can be used, enter the number of indices that will be provided at the next step. If you are planning to use all the expression values, the easiest thing to do is to enter 1 at this stage, and -1 when asked for “expression index”.

*- expression index:*

this is asked repeatedly depending on the number of expression indices entered at the previous stage. Each entry refers to an expression index to apply the threshold check to. If you plan to use all expression values, you should have entered 1 when asked for the “number of expression indices” and you should enter “-1” now.

*- expression threshold value:*

enter the threshold value for expression data

*- keep values above the threshold:*

if the answer is “y”, genes with at least one expression above the threshold are kept. If the answer is “n”, genes with at least one expression below the threshold.are kept.

**============================**

**CLU Creator	(iCluCreator)**

**============================**

**SCHEMA**

**========**

GeneDataFile -----------> iCluCreator ------------> CluFile

**DESCRIPTION**

**============**

This is the main function for creating positional clusters from a set of genes, which must be provided as a GDF file.

Each chromosome is processed separately. Clusters are formed according to the following rule: the gene currently under consideration belongs to the same cluster of the previous one if the distance between the two gene starting points is smaller or equal than a given distance threshold, defined as a multiple of one base-pair (bp), regardless of the strand. If the distance is larger than the threshold, the current gene becomes the first one of a new cluster. As clusters are formed, they are tagged with a progressive, numeric identifier. Clusters formed by only one gene are eliminated during chromosome traversal. The distance threshold is provided (in bp) by the user during the interactive analysis session.

No expression information is used during clustering. It is assumed that any expression-based filtering has already been done in the previous steps.

Cluster information is stored in a text file written in the CLU (cluster file) format, which is structured as follows:

Cluster file (.clu)

nClusters N

1

beginCluster

dset genename chrom strand start stop exp1 exp2 ...

dset genename chrom strand start stop exp1 exp2 ...

dset genename chrom strand start stop exp1 exp2 ...

......

endCluster

2

beginCluster

dset genename chrom strand start stop exp1 exp2 ...

dset genename chrom strand start stop exp1 exp2 ...

........

The total number of clusters (N) is written at the beginning of the file, the clusters are listed in order, preceded by their numeric identifier and delimited by a set of begin-end statements. Within each cluster, the list of contained genes is reported, with complete positional and expression information.

**GUIDE TO OPERATION**

**====================**

From the Matlab prompt, type: "iCluCreator" (enter) and answer the following questions:

*- gdf input file:*

name of the input file containing the genes (GDF format)

*- clu output file:*

name of output file that will contain the cluster (CLU format).

*- minimum threshold distance for clustering:*

numerical value representing the minimum distance (in bp) between two genes that makes them belong to the same cluster.

**=========================**

**CLU Adder	(iCluAdder)**

**=========================**

**SCHEMA**

**========**

```
GeneDataFile ----------->
			           iCluAdder --------------> CluFile
CluFile ---------------->
```

**DESCRIPTION**

**============**

This application offers the possibility to update current clusters with new gene datasets. It is used to identify the behavior of a set of genes when added to existing clusters. The new genes may be incorporated into existing clusters, or they may form new independent clusters. The rules for creating clusters are the same used in iCluCreator and it is recommended that the same distance threshold parameter is used.

The application operates with input data coming from an existing CLU file and a new GDF file containing the additional genes to be processed, the updated clusters are stored into a new CLU file. To be able to distinguish between the “original” genes and the newly added genes in the new CLU file it is convenient to tag the additional genes with a different dataset name in the new GDF file: a typical cluster containing added genes will look as follows in the updated CLU file:

```
beginCluster
dset   genename chrom strand start stop exp1 exp2 ...
dset   genename chrom strand start stop exp1 exp2 ...
dset   genename chrom strand start stop exp1 exp2 ...
dset2  genename chrom strand start stop exp1 exp2 ...
dset2  genename chrom strand start stop exp1 exp2 ...
......
endCluster
```

**GUIDE TO OPERATION**

**====================**

From the Matlab prompt, type “iCluAdder" (enter) and answer the following questions:

*- clu input file:*

name of the input file containing the original clusters (CLU format)

*- gdf input file:*

name of the input file containing the new set of genes (GDF format)

*- clu output file:*

name of the output file that will contain the updated clusters (CLU format)

*- minimum threshold distance for clustering:*

numerical value representing the minimum distance (in bp) between two genes that makes them belong to the same cluster.

**=========================**

**CLU to GDF	(iCluToGdf)**

**=========================**

**SCHEMA SINTETICO:**

**=================**

CluFile ----------------> cluToGdf --------------> gdfFile

**DESCRIPTION**

**============**

This application allows for extracting a single cluster from a CLU file and saving its contents (list of genes) as a GDF file.

**GUIDE TO OPERATION**

**====================**

From the Matlab prompt, type “iCluToGdf" (enter) and answer the following questions:

*- clu input file:*

name of the input file containing the clusters (CLU format)

*- gdf output file:*

name of the output file containing the genes extracted from the clusters (GDF format)

**=========================**

**Cluster Disp (iClusterDisp)**

**=========================**

**SCHEMA**

**========**

CluFile -------> iClusterDisp ----> (cluster contents on screen)

**DESCRIPTION**

**============**

This is a utility application that displays on screen the contents of a single cluster contained in a CLU file.

**GUIDE TO OPERATION**

**====================**

From the Matlab prompt, type “iClusterDisp" (enter) and answer the following questions:

*- clu input file:*

name of the input file containing the clusters (CLU format)

*- cluster index:*

index of the cluster that must be visualized

**=========================**

**Gene Finder	(iGeneFind)**

**=========================**

**SCHEMA**

**========**

CluFile ---------> iGeneFind -------> (cluster index on screen)

**DESCRIPTION**

**============**

This is an utility application that searches for a gene in the clusters, and returns the numerical identifier of the cluster containing the gene (if any). If not found, the application returns -1.

**GUIDE TO OPERATION**

**====================**

From the Matlab prompt, type “iGeneFinder" (enter) and answer the following questions:

*- clu input file:*

name of the input file containing clusters (CLU format)

*- gene name:*

name of the gene to be found in the clusters

**=========================**

**Cluster View (iClusterView)**

**=========================**

**SCHEMA**

**========**

CluFile -----> iClusterView ----> (cluster visualized on figure)

**DESCRIPTION**

**=============**

This application allows for selecting a single cluster from a CLU file and generates a vector-graphics representation that is visualized in a Matlab figure. The representation of the cluster is known as single-cluster view.

**GUIDE TO OPERATION**

**====================**

From the Matlab prompt, type “iClusterView" (enter) and answer the following questions:

*- clu input file:*

name of the input file containing clusters (CLU format)

*- cluster index:*

index of the cluster to be visualized

*- expression visualization mode (circles or diamonds):*

use ‘circles’ to have expressions visualized as circles of varying size, depending on expression values; or use ‘diamonds’ to have diamond-shaped indicators.

- *magnification coefficient for drawing expressions:*

enter the value K so that the size of each circle, or diamond will be roughly proportional to K*expression

**============================**

**All Clusters View (iAllClustersView)**

**============================**

**SCHEMA**

**========**

CluFile --> iAllClustersView ---> (all clusts vis. on figure)

**DESCRIPTION**

**============**

This application allows for visualizing all the clusters contained in a CLU file in a proper vector-graphics format. The format is referred to as multiple-clusters view.

**GUIDE TO OPERATION**

**====================**

From the Matlab prompt, type “iAllClusterView" (enter) and answer the following questions:

*- clu input file:*

name of the input file containing clusters (CLU format)

- *enter 1 to display labels, 0 otherwise:*

enter 1 to visualize cluster label, 0 to hide all the labels (useful for cluttered images with many clusters)

**=================================**

**Random Cluster Creator (iRndCluCreator)**

**=================================**

**SCHEMA**

**========**

GdfFile --> iRndCluCreator ---> (clustering results file)

**DESCRIPTION**

**=============**

This application runs the positional clustering analysis onto sets of randomly selected genes. It is useful for generating data to be used in statistical tests aimed at evaluating the statistical significance of the clustering results obtained for the set of genes being scrutinized.

To produce random clustering data to be used for comparison with actual clustering results for a given set of n selected genes, a GDF file must be prepared containing a larger set of genes (which may or may not include the selected ones). The application reads n genes randomly from the GDF file and runs the clustering analysis. The same clustering process is typically repeated several times, each time randomly selecting a new set of n genes, in order to collect sufficient information for the random sample. Once the process is finished, final clustering data are aggregated and provided as a text file, for being processed, along with the “real” clustering results, in third-party statistics software applications, or within Matlab itself.

The text file is formatted as in the following example:

```
run	n.clusts	n.tot.clust.genes	n.genes per cluster
1	3		6				2 2 2
2	3		7				2 3 2
3	3		6				2 2 2
4	1		2				2
5	3		7				2 2 3
6	2		4				2 2
7	5		13				2 3 2 4 2
```

To read the file, consider for example the fourth row, which refers to the third run (run:3). Three clusters were generated (n.clusts: 3), a total of 6 genes were associated to clusters (n.tot.clust.genes: 6) and each cluster contained exactly 2 genes (n.genes per cluster: 2 2 2).

**GUIDE TO OPERATION**

**====================**

From the Matlab prompt, type “iRndCluCreator" (enter) and answer the following questions:

*- gdf input file:*

name of the input file containing the genes (GDF format). Note that expression data will not be used.

*- output file:*

name of the output file containing the summary of the clustering results for all the programmed runs.

*- minimum threshold distance for clustering:*

minimum distance between genes (in bp) so that they can be considered as belonging to the same cluster.

*- how many genes to extract random from GDF file at each run?*

Number of genes that will be randomly extracted from the GDF file for each clustering run.

*- how many times you want random clustering to be run?*

Number ot times the random clustering procedure will be repeated

**=================================**

**All Clusters Analyze (iAllClustersAnalyze)**

**=================================**

**SCHEMA**

**========**

CluFile --> iAllClustersAnalyzerView ---> (cluster analysis file)

**DESCRIPTION**

**============**

This is a simple utility function that provides quantitative information concerning the contents of a CLU file. More specifically, it returns the probability distribution of cluster sizes, written as a text file.

**GUIDE TO OPERATION**

**====================**

From the Matlab prompt, type “iAllClustersAnalyze" (enter) and answer the following questions:

*- clu input file:*

name of the input file containing clusters (CLU format)

- *txt output file:*

name of the text file that will contain the analysis results
